# Isobutanol Production by Autotrophic Acetogenic Bacteria

**DOI:** 10.3389/fbioe.2021.657253

**Published:** 2021-04-12

**Authors:** Sandra Weitz, Maria Hermann, Sonja Linder, Frank R. Bengelsdorf, Ralf Takors, Peter Dürre

**Affiliations:** ^1^Institut für Mikrobiologie und Biotechnologie, Universität Ulm, Ulm, Germany; ^2^Institut für Bioverfahrenstechnik, Universität Stuttgart, Stuttgart, Germany

**Keywords:** *Acetobacterium woodii*, acetogens, *Clostridium ljungdahlii*, gas fermentation, isobutanol production, syngas

## Abstract

Two different isobutanol synthesis pathways were cloned into and expressed in the two model acetogenic bacteria *Acetobacterium woodii* and *Clostridium ljungdahlii*. *A. woodii* is specialized on using CO_2_ + H_2_ gas mixtures for growth and depends on sodium ions for ATP generation by a respective ATPase and Rnf system. On the other hand, *C. ljungdahlii* grows well on syngas (CO + H_2_ + CO_2_ mixture) and depends on protons for energy conservation. The first pathway consisted of ketoisovalerate ferredoxin oxidoreductase (Kor) from *Clostridium thermocellum* and bifunctional aldehyde/alcohol dehydrogenase (AdhE2) from *C. acetobutylicum*. Three different *kor* gene clusters are annotated in *C. thermocellum* and were all tested. Only in recombinant *A. woodii* strains, traces of isobutanol could be detected. Additional feeding of ketoisovalerate increased isobutanol production to 2.9 mM under heterotrophic conditions using *kor*3 and to 1.8 mM under autotrophic conditions using *kor*2. In *C. ljungdahlii*, isobutanol could only be detected upon additional ketoisovalerate feeding under autotrophic conditions. *kor*3 proved to be the best suited gene cluster. The second pathway consisted of ketoisovalerate decarboxylase from *Lactococcus lactis* and alcohol dehydrogenase from *Corynebacterium glutamicum*. For increasing the carbon flux to ketoisovalerate, genes encoding ketol-acid reductoisomerase, dihydroxy-acid dehydratase, and acetolactate synthase from *C. ljungdahlii* were subcloned downstream of *adhA*. Under heterotrophic conditions, *A. woodii* produced 0.2 mM isobutanol and 0.4 mM upon additional ketoisovalerate feeding. Under autotrophic conditions, no isobutanol formation could be detected. Only upon additional ketoisovalerate feeding, recombinant *A. woodii* produced 1.5 mM isobutanol. With *C. ljungdahlii*, no isobutanol was formed under heterotrophic conditions and only 0.1 mM under autotrophic conditions. Additional feeding of ketoisovalerate increased these values to 1.5 mM and 0.6 mM, respectively. A further increase to 2.4 mM and 1 mM, respectively, could be achieved upon inactivation of the *ilvE* gene in the recombinant *C. ljungdahlii* strain. Engineering the coenzyme specificity of IlvC of *C. ljungdahlii* from NADPH to NADH did not result in improved isobutanol production.

## Introduction

**FIGURE 1:**
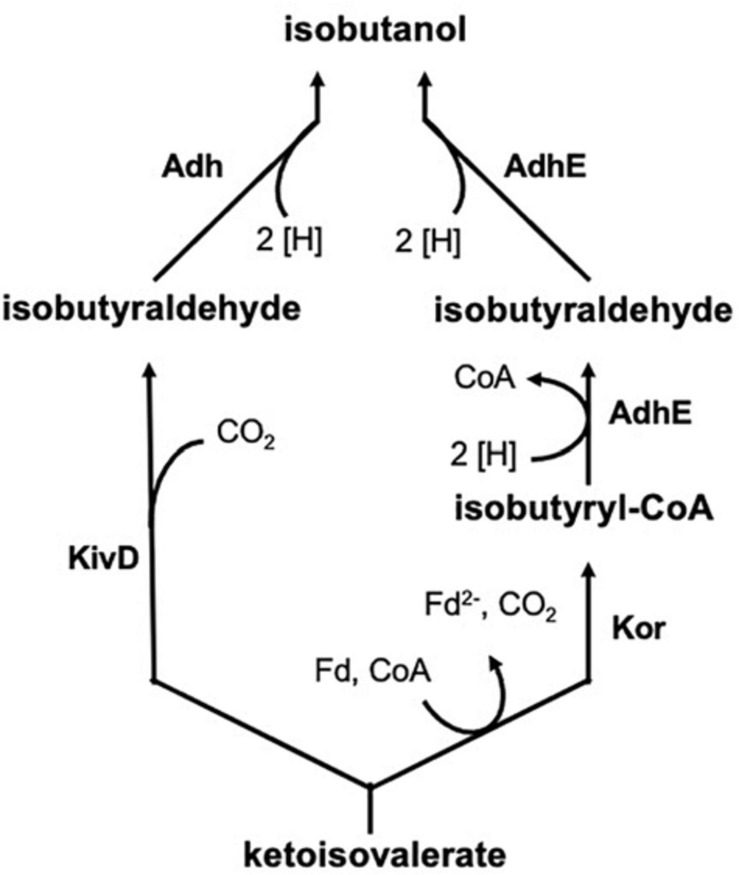
Formation of isobutanol by the enzymes KivD (ketoisovalerate decarboxylase) and Adh (alcohol dehydrogenase) or Kor (ketoisovalerate ferredoxin oxidoreductase) and AdhE (bifunctional aldehyde/alcohol dehydrogenase) from ketoisovalerate, the precursor of valine.

Isobutanol is an important platform chemical, representing an app. one billion US $ market in 2019. The compound is mainly used in solvents and coatings, as a chemical intermediate, or as a biofuel (gasoline additive). Synthesis is chemically achieved by hydroformylation of propylene, followed by hydrogenation of the formed aldehyde, but bio-based processes have been developed by companies Gevo Inc. (Douglas County, CO) and Butamax Advanced Biofuels LLC (Wilmington, DE; joint venture of BP and DuPont). The share of bio-based isobutanol is expected to rise in the near future significantly compared to the synthetic compound. A number of microorganisms have been engineered for isobutanol production, including aerobic and anaerobic bacteria as well as *Saccharomyces cerevisiae* (Chen and Liao, 2016). Gevo is using a recombinant yeast for its corn-based process, but details have not yet been disclosed. A landmark publication reported genetically engineered isobutanol formation by modification of amino acid synthesis pathways (Atsumi et al., 2008). 2-Ketoisovalerate, an intermediate in valine and isoleucine biosynthesis, was first decarboxylated by e.g., KivD from *Lactococcus lactis* and then the formed aldehyde was reduced to isobutanol by an alcohol dehydrogenase (e.g., Adh2 from *Saccharomyces cerevisiae*) (Figure 1). A natural pathway for production is based on the enzyme ketoisovalerate ferredoxin oxidoreductase (Kor), which was first found and characterized in hyperthermophilic archaea, where it is involved in branched-chain amino acid degradation and biosynthesis (Heider et al., 1996). This isobutanol synthesis pathway (Figure 1) was shown to be active in e.g., *Clostridium thermocellum* (Lin et al., 2015).

Using biological production with recombinant strains, high isobutanol titers in typical fermentation setups were obtained with e.g., *E. coli* (22 g/l; Atsumi et al., 2008), *Corynebacterium glutamicum* (13 g/l; Blombach et al., 2011), *Clostridium thermocellum* (5.4 g/l; Lin et al., 2015), *Geobacillus thermoglucosidasius* (3.3 g/l; Lin et al., 2014), *S. cerevisiae* (2.1 g/l; Wess et al., 2019), and *Ralstonia eutropha* (0.03 g/l; Black et al., 2018). All these processes used sugar or cellulose as a carbon source. Cheaper substrates would be CO_2_ (in combination with a reductant) or CO, which are also waste and greenhouse gases. A photosynthetic isobutanol production of up to 0.9 g/l from CO_2_ and solar energy has been reported for *Synechocystis* PCC 6803 (Miao et al., 2018). The electrochemical conversion of CO_2_ and water to formate was coupled with formate-dependent growth of recombinant cells of the aerobic “Knallgas” bacterium *R. eutropha*, yielding up to 0.85 g isobutanol/l (Li et al., 2012). However, some challenges still remain for CO_2_-based biological isobutanol formation compared to the non-biological production (Brigham, 2019).

Here, we report an approach to isobutanol synthesis using two anaerobic bacteria, which became model organisms of the so-called acetogens: *Acetobacterium woodii*, growing on CO_2_/H_2_, and *Clostridium ljungdahlii*, growing on syngas (a mixture of mostly CO and H_2_).

## Materials and Methods

### Bacterial Strains and Plasmids

*Acetobacterium woodii* (DSM 1030) and *Clostridium ljungdahlii* (DSM13583) were obtained from the DSMZ (Deutsche Sammlung von Mikroorganismen und Zellkulturen GmbH, Brunswick, Germany). *Escherichia coli* XL1-Blue MRF’ [Δ*(mcrA)183*Δ(*mcrCB-hsdSMR-mrr*)*173 endA1 supE44 thi-1*] stemmed from Stratagene (La Jolla, CA). All plasmids used in this study are listed in Table 1.

**TABLE 1:** Plasmids used in this study.

**Plasmid**	**Relevant features**	**References**
pJUL34	*kivD* from *L. lactis*, P_tuf_ from *L. lactis*, *adhA* from *Corynebacterium glutamicum*; *aph3*; *sacB*, replicon repBL1 for *Corynebacterium*	Gift from Bastian Blombach (Technical University of Munich, Germany) and Bernhard Eikmanns (University of Ulm, Germany)
pMTL007-E2_ilvE	ColE1 ori^–^, pCB102 ori^+^, *catP*, *lacZ*, *ltrA*, *oriT*, Pfdx, *ermB*, *traJ*, ClosTron^TM^plasmid to inactivate *ilvE* in the genome of *C. ljungdahlii*	This study, ATUM, Newark, CA, United States
pMTL83151	*catP*, ColE1 ori^–^, *lacZ*, pCB102 ori^+^, *traJ*	Heap et al., 2009
pMTL83151_ ptaack_aacht_cac	pMTL83151, P_pta–ack_ and *adhE2* from *C. acetobutylicum*; *abfD* from *C. scatologenes*, *crt*, *hbd*, and *thlA* from *C. acetobutylicum*	This study
pKAIA	pMTL83151, P_pta–ack_ from *C. ljungdahlii, kivD* from *L. lactis*, *adhA* from *Corynebacterium glutamicum*, *ilvC*, *ilvD*, and *alsS* from *C. ljungdahlii*	This study
pKAI_NADH_A	pMTL83151 P_pta–ack_ from *C. ljungdahlii*, *kivD* from *L. lactis, adhA* from *C. glutamicum*, *ilvD* and *alsS* from *C. ljungdahlii*, ilvC^*P2D1–A1*^ from *E. coli*, codon optimized for clostridia	This study, Thermo Fisher Scientific GENEART GmbH, Regensburg, Germany, Brinkmann-Chen et al., 2013
pKOR2	pMTL83151, P_pta–ack_ from *C. ljungdahlii*, *adhE2* from *C. acetobutylicum*, *kor2* from *C. thermocellum*	This study
pKOR3	pMTL83151, P*_*pta–ack*_* from *C. ljungdahlii*, *adhE2* from *C. acetobutylicum*, *kor3* from *C. thermocellum*	This study

### Media and Growth Conditions

*Escherichia coli* was cultivated aerobically in LB (Luria-Bertani) medium (per l: tryptone 10 g, yeast extract 5 g, NaCl 10 g) (Green and Sambrook, 2012) at 37°C on a rotary shaker (175 rpm). For generation of competent cells, *E. coli* was grown in modified SOB (Super Optimal Broth; per l: tryptone 20 g, yeast extract 5 g, NaCl 0.5 g, KCl 1.92 g, MgCl_2_ × 6 H_2_O 2.03 g) (Green and Sambrook, 2012).

Basal medium for *A. woodii* was modified from Balch et al. (1977) and contained per l: KH_2_PO_4_ 0.33 g, K_2_HPO_4_ 0.45 g, MgSO_4_ × 7 H_2_O 0.33 g, NH_4_Cl 1 g, yeast extract 2 g, L-cysteine-HCl × H_2_O 0.5 g, resazurin 1 mg. To this solution, 20 ml of trace element solution (per l: nitrilotriacetic acid 1.5 g, MgSO_4_ × 7 H_2_O 3 g, MnSO_4_ × H_2_O 0.5 g, NaCl 1 g, FeSO_4_ × 7 H_2_O 0.1 g, CoSO_4_ × 7 H_2_O 0.18 g, CaCl_2_ × 2 H_2_O 0.1 g, ZnSO_4_ × 7 H_2_O 0.18 g, CuSO_4_ × 5 H_2_O 0.01 g, KAl(SO_4_)_2_ × 12 H_2_O 0.02 g, H_3_BO_3_ 0.01 g, Na_2_MoO_4_ × 2 H_2_O 0.01 g, NiCl_2_ × 6 H_2_O 0.03 g, Na_2_SeO_3_ × 5 H_2_O 0.3 mg, Na_2_WO_4_ × 2 H_2_O 0.4 mg) and 20 ml vitamin solution (per l: biotin 2 mg, folic acid 2 mg, pyridoxine-HCl 10 mg, thiamine-HCl 5 mg, riboflavin 5 mg, nicotinic acid 5 mg, D-Ca-pantothenate 5 mg, vitamin B_12_ 0.1 mg, p-aminobenzoic acid 5 mg, lipoic acid 5 mg (DSMZ_Medium141-1.pdf,^1^) were added. Media were prepared under strictly anaerobic conditions. Heterotrophic growth was performed with 40 mM fructose as a carbon source under an atmosphere consisting of N_2_ (80%) and CO_2_ (20%), autotrophic growth with a CO_2_ + H_2_ gas mixture (33% + 67%), both at 30°C.

*Clostridium ljungdahlii* was cultivated in a modified medium described by Tanner et al. (1993). It contained per l: 2-(*N*-morpholino) ethanesulfonic acid 20 g, yeast extract 0.5 g, L-cysteine-HCl × H_2_O 1 g, resazurin 1 mg. To this solution, 25 ml of mineral salts solution (per l: CaCl_2_ × 2 H_2_O 4 g, KCl 10 g, KH_2_PO_4_ 10 g, MgSO_4_ × 7 H_2_O 20 g, NaCl 80 g, NH_4_Cl 100 g), 10 ml of modified trace element solution (American Type Culture Collection (ATCC);^2^; 1754 PETC medium) (per l: nitrilotriacetic acid 2 g, MnSO_4_ × H_2_O 0.5 g, Fe(SO_4_)_2_(NH_4_)_2_ × 6 H_2_O 0.8 g, CoCl_2_ × 6 H_2_O 0.2 g, ZnSO_4_ × 7 H_2_O 1 mg, CuCl_2_ × 2 H_2_O 20 mg, NiCl_2_ × 6 H_2_O 20 mg, Na_2_MoO_4_ × 2 H_2_O 20 mg, Na_2_SeO_3_ × 5 H_2_O 20 mg, Na_2_WO_4_ × 2 H_2_O 20 mg), and 10 ml vitamin solution (per l: biotin 2 mg, folic acid 2 mg, pyridoxine-HCl 10 mg, thiamine-HCl 5 mg, riboflavin 5 mg, nicotinic acid 5 mg, D-Ca-pantothenate 5 mg, vitamin B_12_ 5 mg, p-aminobenzoic acid 5 mg, and lipoic acid 5 mg) were added. Media were prepared under strictly anaerobic conditions. Heterotrophic growth was performed with 40 mM fructose as a carbon source under an atmosphere consisting of N_2_ (80%) and CO_2_ (20%), autotrophic growth with a syngas mixture (50% CO, 45% H_2_, and 5 CO_2_), both at 37°C.

For cultivation of *A. woodii* and *C. ljungdahlii* on solid media, a modified version of yeast tryptone fructose medium (Leang et al., 2013) was used (per l: yeast extract 10 g, tryptone 16 g, fructose 5 g, NaCl 4 g, L-cysteine-HCl × H_2_O 1 g, resazurin 1 mg, agar 15 g). Agar plates were poured using anaerobic water in an anaerobic cabinet containing a N_2_:H_2_ (95:5%) gas atmosphere. Cell suspensions were dripped on plates and spread using glass beads. *A. woodii* cells were incubated at 30°C and *C. ljungdahlii* cells at 37°C in an incubator located in the anaerobic cabinet.

For selection of antibiotic-resistant strains, the following concentrations were used per ml: ampicillin 100 mg, clarithromycin 2.5 mg, chloramphenicol 30 mg, kanamycin 50 mg, thiamphenicol 7.5 mg.

### Analytical Methods

Cell growth was monitored offline by measuring the optical density at 600 nm (Ultrospec 3100, Amersham Bioscience Europe GmbH, Freiburg, Germany) in 1-ml cuvettes (width 1 cm).

Ethanol, isobutanol, and isoamyl alcohol were determined using gas chromatograph (GC) “Clarus 680” (PerkinElmer, PerkinElmer, Waltham, MA, United States). GC was equipped with an Elite-FFAP column (i ∅ 0.32 mm × 30 m). H_2_ was the carrier gas (2.25 ml min^–1^, 100°C), injector temperature was 225°C, split 1:20, and flame ionization detector temperature was 300°C. Detector gases were synthetic air (450 ml min^–1^) and H_2_ (45 ml min^–1^). A temperature profile was predefined: 90°C for 2 min, 40°C min^–1^ increasing steps to 250°C (constant for 1 min). Supernatant (0.48 ml) was acidified with 0.02 mL of 2 M HCl. 1 ml was injected into the GC. Calibration was performed using defined standards of the individual components.

Acetate, 2,3-butanediol, fructose, and ketoisovalerate were determined using high performance liquid chromatography (Agilent 1260 Infinity Series HPLC, Agilent Technologies, Santa Clara, CA) equipped with a refractive index detector (for 2,3-butanediol, fructose, ketoisovalerate) operating at 35°C and a diode array detector (for acetate) at room temperature. The “CS-Chromatographie organic acid column” (CS-Chromatographie Service GmbH, Langerwehe, Germany) was kept at 40°C. 5 mM H_2_SO_4_ was used as mobile phase with a flow rate of 0.7 ml min^–1^. 20 μl of supernatant were injected into the HPLC system for determination of compounds.

### DNA Isolation

Bacterial genomic DNA was isolated using the “MasterPure^TM^ Gram-Positive DNA Purification Kit” (Epicentre, Madison, WI, United States). 2-mL Samples of late exponential cultures were centrifuged (18,000 g, 30 min, 4°C) and further processed according to the manufacturer’s instructions. Isolation of plasmid DNA from *E. coli* strains was performed with the “Zyppy^TM^ Plasmid Miniprep Kit” (ZYMO Research Europe GmbH, Freiburg, Germany). 4-ml Samples of overnight cultures were centrifuged (18,000 g, 1 min) and further processed according to the manufacturer’s instructions. In case of *A. woodii* and *C. ljungdahlii*, 2 ml were sampled and centrifuged (18,000 g, 30 min, 4°C). The cell pellet was suspended in 600 μl Tris−HCl (20 mM, pH 7). For effective lysis, 60 μl lysozyme (20 mg ml^–1^) were added, and the solution was incubated for 1 h at 37°C before proceeding according to the manufacturer’s instructions.

DNA from *C. thermocellum* DSM 1313 was purchased from the DSMZ (Deutsche Sammlung von Mikroorganismen und Zellkulturen GmbH, Brunswick, Germany).

Sequencing of DNA was performed by GATC Biotech AG (Constance, Germany).

### Plasmid Construction

Standard molecular cloning techniques were performed according to established protocols (Green and Sambrook, 2012). In *C. thermocellum*, three gene clusters encoding a putative ketoisovalerate ferredoxin oxidoreductase (Kor) are annotated [*kor1* (Clo1313_0020-0023), *kor2* (Clo1313_0382-0385), *kor3* (Clo1313_1353-1356)]. *kor1* was PCR-amplified using primers clo0020T_fwd and clo0020T_rev, *kor2* was PCR-amplified using primers clo0382T_fwd and clo0382T_rev, and *kor3* was PCR-amplified using primers clo1353T_fwd and clo1353T_rev. Then, each fragment was subcloned into the pMTL83151 backbone (Heap et al., 2009), together (upstream) with the also PCR-amplified P_*pta–ack*_ promoter from *C. ljungdahlii* (primers: Ptaack_fwd and Ptaack_rev) and the gene encoding bifunctional butyraldehyde/butanol dehydrogenase AdhE2 from *C. acetobutylicum* [primers: adhE2_pka_fwd and adhE2_pka_rev; plasmid pMTL83151_ptaack_aacht_cac (Supplementary Figure 1)]. All primers used are listed in Table 2. Subcloning was performed by in-fusion cloning using the NEBuilder Assembly Tool (New England Biolabs Inc., Ipswich, MA, United States;^3^). The resulting plasmids pKOR1, pKOR2, and pKOR3 are shown in Supplementary Figure 2. pMTL83151 was kindly provided by Nigel Minton (University of Nottingham, United Kingdom).

**TABLE 2:** Primer used for cloning procedures.

**Name**	**Sequence**	**Amplification**
clo0020T_fwd	GATATCTATATAAAATCATTTTAACCTCG AGAGGAGGATTACCACATGGGCAA	KOR1 Clo1313_0020-0023
clo0020T_rev	CAAATGCAGGCTTCTTATTTTTATGGCTA GCCAATAATATTTTCTCATTTTAAAAAAT	
clo0382T_fwd	GATATCTATATAAAATCATTTTAACCTCG AGAGGGAGCGATGGAGATGACAGA	KOR2 Clo1313_0382-0385
clo0382T_rev	CAAATGCAGGCTTCTTATTTTTATGGCTA GCCTCCTTTTTTCAAAAAAAGTCCATGTT CC	
clo1353T_fwd	GATATCTATATAAAATCATTTTAACCTCG AGGGGGGATTTACATGGCTAAGGT	KOR3 Clo1313_1353-1356
clo1353T_rev	CAAATGCAGGCTTCTTATTTTTATGGCTA GCCGTATAAGAATTAAAAATAAGAATTA AAAATCAAAACAATCAAAAAAAG	
Ptaack_fwd	ACAGCGGCCGCGTCGACGTTACCACTCAT	P*_*pta–ack*_*
Ptaack_rev	ACAACCGGTCCTCAGGTCCTCCCTTTA	
adhE2_pka_fwd	TTAAATTTAAAGGGAGGACCTGAGGATG AAAGTTACAAATCAAAAAGAAC	*adhE2*
adhE2_pka_rev	CTTGGGGTGCAGCAGTGGTCATCCTCGAG TTAAAATGATTTTATATAGATATCCTTAA GTTC	
kivd_fwd_XhoI	CTCGAGGTCCTCCTATTTATAAATTATG	*kivD*
kivd_rev_Eco81I	ACACCTGAGGATGTATACAGTAGGAG	
adhA_fwd_XhoI	CTCGAGATGACCACTGCTGCACCC	*adhA*
adhA_rev_NheI	ACAGCTAGCGCGAGTCGAACAGATGTG	
ilvC_fwd	TGTGGCGATTCGTTTCTAACGACGTCAAA ATAGTATAAATAAATTATTCAGGAGG	*ilvC*
ilvC_rev	TGTAAAAAAATACTAGTTTACTCATTATC AGGATTTTCATTG	
ilvD_fwd	TAATGAGTAAACTAGTATTTTTTTACAAA AAATTTCCAG	*ilvD*
ilvD_rev	GATCTTTATACCATGGTTATTTAAGAACT GCACCTGTATTTG	
alsS_fwd	TTCTTAAATAACCATGGTATAAAGATCAG AGGAAGTTTTATATG	*alsS*
alsS_rev	CAAAGTAGCTTCAGAGCAGTTCTAGATTA CATATTTTCATAAACTTCTTTTAAATG	

*Underlined nucleotides are vector overlapping fragments used for In-Fusion^®^ cloning.*

The plasmids constructed for elucidation of the ketoisovalerate decarboxylase pathway were also based on the pMTL83151 backbone. The *kivD* gene from *L. lactis* was PCR-amplified using primers kivd_fwd_XhoI and kivd_rev_Eco81I (making use of the inserted restriction sites for subcloning), *adhA* from *C. glutamicum* was PCR-amplified using primers adhA_fwd_XhoI and adhA_rev_NheI (making use of the inserted restriction sites for subcloning). DNA source for amplification was plasmid pJUL34 (Supplementary Figure 3), which was kindly provided by Bastian Blombach (Technical University of Munich, Germany) and Bernhard Eikmanns (University of Ulm, Germany) (Table 1). Both fragments were subcloned together in several steps into pMTL83151. Upstream of *kivD*, the PCR-amplified P_pta–ack_ promoter fragment from *C. ljungdahlii* (primers: Ptaack_fwd and Ptaack_rev) was inserted. The genes *ilvC* (encoding ketol-acid reductoisomerase) (primers ilvC_fwd and ilvC_rev), *ilvD* (encoding dihydroxy-acid dehydratase) (primers ilvD_fwd and ilvD_rev), and *alsS* (encoding acetolactate synthase) (primers alsS_fwd and alsS_rev), all from *C. ljungdahlii*, were PCR-amplified (Table 2). Subcloning downstream of *adhA* was performed by in-fusion cloning using the NEBuilder Assembly Tool (New England Biolabs inc., Ipswich, MA, United States; see text footnote 3). The resulting plasmid was designated pKAIA (Supplementary Figure 4).

For changing the coenzyme specificity of IlvC (ketol-acid reductoisomerase) of *C. ljungdahlii* from NADPH to NADH, the ilvC^*NADH*^ gene of *E. coli* was commercially synthesized according to Brinkmann-Chen et al. (2013), with appropriate overlaps for in-fusion cloning. Codon optimization for clostridia was suggested by the commercial supplier Thermo Fisher Scientific GENEART GmbH (Regensburg, Germany). The sequence of the codon-optimized, synthesized gene is provided in Supplementary Figure 5.

The *ilvC* gene was cut out of pKAIA and the codon-optimized, synthesized gene was subcloned into the linearized vector using in-fusion cloning, resulting in plasmid pKAI_NADH_A (Supplementary Figure 6).

The ClosTron plasmid pMTL007-E2_ilvE (Table 1) was synthesized by the company DNA 2.0 (now ATUM, Newark, CA, United States) (see below).

### Transformation

In case of *E. coli*, competent cells (Inoue et al., 1990) were obtained by inoculating 250 ml SOB medium with a 5-ml overnight culture grown in the same medium. Incubation was performed in a 2-l Erlenmeyer flask at 18°C under aerobic conditions and shaking (60 rpm), until an optical density (600 nm) of 0.6–0.8 was reached. Then, cells were put on ice for 30 min and afterward centrifuged (4,500 g, 4°C, 10 min). The pellet was suspended in 40 ml buffer (per 125 ml: piperazine-N,N’–bis(2-ethanesulfonic acid) 0.756 g, CaCl_2_ 0.42 g, pH 6.7; mixed with 125 ml containing 4.66 g KCl and 1.72 g MnCl_2_ × 4 H_2_O), incubated on ice for 10 min, and again centrifuged (4,500 g, 4°C, 10 min). The pellet was suspended in 10 ml of the same buffer. 1.5 ml sterile dimethyl sulfoxide were added, and the solution was split into 200 μl aliquots and stored at −80°C. For transformation, such aliquots were thawed on ice, mixed with 10 μl of a DNA solution (in-fusion reaction mix or plasmid), incubated on ice for 10 min, and then at 42°C for 1 min. After an incubation period of 10 min again on ice, 800 μl LB medium were added, and the solution was incubated aerobically for 1 h at 37°C with shaking (160 rpm). Then, cells were centrifuged (4,000 g, room temperature, 3 min), and 800 μl of the supernatant discarded. The pellet was resuspended in the remaining liquid, which was used for plating on solid media with respective selection.

Electroporation of *A. woodii* and *C. ljungdahlii* was performed using a modified protocol of Leang et al. (2013). All plastic materials were stored in an anaerobic cabinet at least 1 day before transformation to eliminate any remaining traces of oxygen. For preparation of competent cells, 100 ml medium were supplemented with 40 mM fructose and 40 mM DL-threonine, inoculated with an early exponential culture, and grown at the appropriate temperature to an optical density (600 nm) of 0.3–0.7. Cultures were centrifuged anaerobically (6,000 g, 4°C, 10 min), and the pellet was washed twice with 50 ml cool, anaerobic SMP buffer (per l: sucrose 92.4 g, MgCl_2_ × 6 H_2_O 0.2 g, NaH_2_PO_4_ 0.84 g; pH 6) and suspended in 0.6 ml SMP buffer. 120 μl cool anti-freezing buffer (mix of 20 ml SMP buffer and 30 ml dimethylformamide) were added, and aliquots stored at −80°C.

Electroporation was carried out in an anaerobic cabinet. 25 μl of electrocompetent cells were mixed with 3–5 μg of plasmid DNA, cooled, and transferred to a pre-cooled 1-mm gap electroporation cuvette (Biozym Scientific GmbH, Hessisch Oldendorf, Germany). Electric pulse was performed with 625 V, resistance of 600 Ω, and a capacitance of 25 μF using a “Gene-Pulser Xcell^TM^” pulse generator (Bio-Rad Laboratories GmbH, Munich, Germany). Afterward, cells were recovered using 5 ml of the respective medium without antibiotic in a Hungate tube and incubated until the optical density (600 nm) doubled. Then, the appropriate antibiotic was added and growth monitored. If growth was detectable, cells were inoculated into fresh medium with antibiotic and, after reaching the early exponential growth phase, plated onto solid media. Single colonies of obtained transformants were picked, and successful transformation was confirmed by isolating genomic or plasmid DNA and PCR amplification of respective sequences for verification.

### Gene Inactivation by ClosTron

The ClosTron method was performed according to Heap et al. (2007, 2010). The aim was to create an insertion mutant in *ilvE* of *C. ljungdahlii*, which blocked the last step of valine biosynthesis. Target region for integration was determined using the respective algorithm (Perutka et al., 2004;^4^), and the respective ClosTron plasmid (pMTL007-E2_ilvE) (Table 1) was synthesized by the company DNA 2.0 (now ATUM, Newark, CA). After transformation into *C. ljungdahlii*, recombinant strains were verified by PCR amplification and sequencing of the respective gene region.

## Results

### Isobutanol Production *via* Ketoisovalerate Ferredoxin Oxidoreductase (Kor)

**FIGURE 2:**
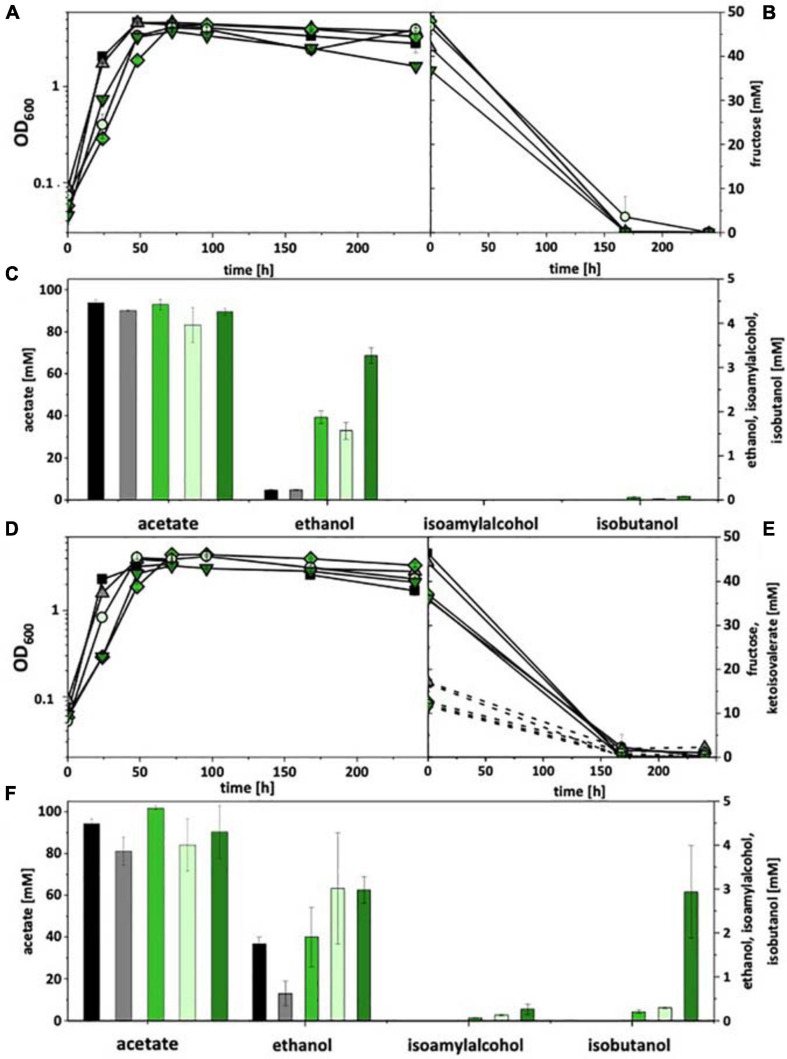
Heterotrophic isobutanol production with recombinant *A. woodii* strains by the Kor pathway. Growth behavior **(A,D)**; fructose consumption **(B,E)**; ketoisovalerate consumption **(E)**; product pattern **(C,F)**. *A. woodii* [WT], black, squares; *A. woodii* [pM83], gray, triangles; *A. woodii* [pKOR1], green, diamonds; *A. woodii* [pKOR2], light green, circles; *A. woodii* [pKOR3], dark green, triangle pointing downward. Panels **(A–C)** without ketoisovalerate supplementation; Panels **(D–F)** with ketoisovalerate supplementation. Each strain was analyzed in biological triplicates (*n* = 3).

In the genome of *C. thermocellum* three gene clusters are annotated that encode a putative ketoisovalerate ferredoxin oxidoreductase (Kor), i.e., *kor1* (Clo1313_0020-0023), *kor2* (Clo1313_0382-0385), and *kor3* (Clo1313_1353-1356). *kor3* differs significantly from the other two clusters with respect to length (smaller) and gene arrangement (Supplementary Figure 7). However, all clusters consist of four genes, encoding the α, β, γ, and δ subunits of Kor. The α subunit carries the core domain of pyruvate-ferredoxin oxidoreductase, β a C-terminal thiamine pyrophosphate-binding domain, γ a catalytic domain of pyruvate/ketoisovalerate oxidoreductase, and δ a binding domain for 4Fe-4S ferredoxin. All clusters were PCR-amplified and subcloned each into the pMTL83151 backbone, together (upstream) with the P_pta–ack_ promoter from *C. ljungdahlii* and the gene encoding bifunctional butyraldehyde/butanol dehydrogenase AdhE2 from *C. acetobutylicum*. The resulting plasmids (pKOR1, pKOR2, and pKOR3) were transformed into *A. woodii* and *C. ljungdahlii*. Recombinant strains were then tested under heterotrophic conditions with fructose as a carbon source as well as with and without addition of ketoisovalerate (15 mM). Both, *A. woodii* and *C. ljungdahlii* did not grow with ketoisovalerate as sole carbon and energy source and also did not show an increase in optical density when fructose and ketoisovalerate were supplied together, compared to fructose alone. Wild type strains and empty plasmid-carrying strains [*A. woodii* (pM83) and *C. ljungdahlii* (pM83)] were used as controls. Without ketoisovalerate addition, recombinant *A. woodii* strains only formed traces of isobutanol (0.1 mM). However, all pKOR-carrying strains produced increased amounts of ethanol. With addition of ketoisovalerate, *A. woodii*[pKOR1] produced 0.2 mM isobutanol, *A. woodii*[pKOR2] 0.3 mM, and *A. woodii*[pKOR3] 2.9 mM (Figure 2). Some isoamyl alcohol (up to 0.3 mM) was formed in addition. The respective data for *C. ljungdahlii* are shown in Supplementary Figure 8. With and without ketoisovalerate addition, no isobutanol was formed. pKOR-carrying strains produced increased amounts of ethanol with addition of ketoisovalerate.

**FIGURE 3:**
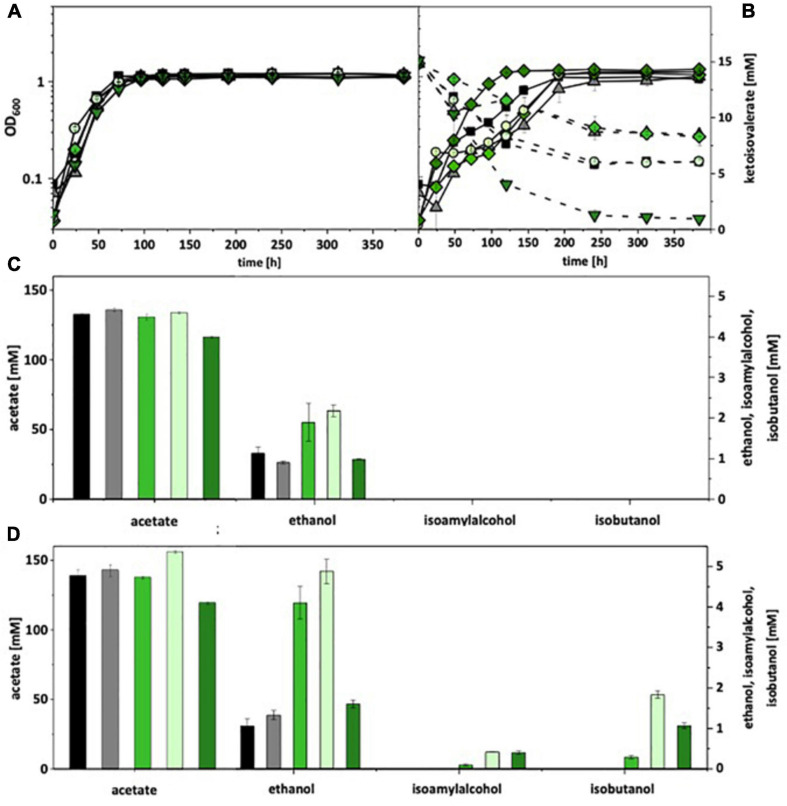
Autotrophic isobutanol production with recombinant *A. woodii* strains by the Kor pathway. Growth behavior **(A,B)**; ketoisovalerate consumption **(B)**; product pattern **(C,D)**. *A. woodii* [WT], black, squares; *A. woodii* [pM83], gray, triangles; *A. woodii* [pKOR1], green, diamonds; *A. woodii* [pKOR2], light green, circles; *A. woodii* [pKOR3], dark green, triangle pointing downward. Panels **(A,C)** without ketoisovalerate supplementation; Panels **(B,D)** with ketoisovalerate supplementation. Each strain was analyzed in biological triplicates (*n* = 3).

For autotrophic growth, *A. woodii* was cultivated under a CO_2_ + H_2_ atmosphere, *C. ljungdahlii* under syngas. Without ketoisovalerate addition, recombinant *A. woodii* strains formed no isobutanol. With addition, *A. woodii*[pKOR1] produced 0.3 mM, *A. woodii*[pKOR2] 1.8 mM, and *A. woodii*[pKOR3] 1.1 mM (Figure 3). *A. woodii*[pKOR1] and *A. woodii*[pKOR2] produced also increased amounts of ethanol. Some isoamyl alcohol (up to 0.4 mM) was formed in addition. The respective data for *C. ljungdahlii* are shown in Supplementary Figure 9. Without ketoisovalerate addition, no isobutanol was formed and even with addition only trace amounts [*C. ljungdahlii*(pKOR2) 0.1 mM; *C. ljungdahlii*(pKOR3) 0.2 mM]. However, *C. ljungdahlii*[pKOR3] showed a significant increase in ethanol formation without ketoisovalerate addition as well as *C. ljungdahlii*[pKOR2] with addition.

### Isobutanol Production *via* Ketoisovalerate Decarboxylase (KivD)

Next, the isobutanol synthesis pathway *via* ketoisovalerate decarboxylase and alcohol dehydrogenase in acetogens was elucidated. The *kivD* gene from *L. lactis* and *adhA* from *C. glutamicum* were PCR-amplified and subcloned together into the pMTL83151 backbone, controlled by the also PCR-amplified P_pta–ack_ promoter from *C. ljungdahlii*. In order to increase the carbon flux from pyruvate to ketoisovalerate, genes *ilvC* (encoding ketol-acid reductoisomerase), *ilvD* (encoding dihydroxy-acid dehydratase), and *alsS* (encoding acetolactate synthase), all from *C. ljungdahlii*, were PCR-amplified and subcloned downstream of *adhA*. The resulting plasmid pKAIA was transformed into *A. woodii* and *C. ljungdahlii*. In case of *C. ljungdahlii*, it was also possible to construct an insertion mutant in *ilvE* (encoding an aminotransferase), which blocked the last step of valine biosynthesis. This strain, *C. ljungdahlii:ilvE* was also transformed with pKAIA. Recombinant strains were then tested under heterotrophic conditions with fructose as a carbon source as well as with and without addition of ketoisovalerate (15 mM). Wild type strains and empty plasmid-carrying strains were used as controls. Without ketoisovalerate addition, only *A. woodii*[pKAIA] formed some isobutanol (0.2 mM). With addition, the same strain produced 0.4 mM (Supplementary Figure 10). Some isoamyl alcohol (up to 0.2 mM) was formed in addition. The respective data for *C. ljungdahlii* are shown in Supplementary Figure 11. Without ketoisovalerate addition, no isobutanol was formed. With addition, *C. ljungdahlii*[pKAIA] and *C. ljungdahlii:ilvE*[pKAIA] produced up to 2.4 mM isobutanol during the exponential growth phase.

**FIGURE 4:**
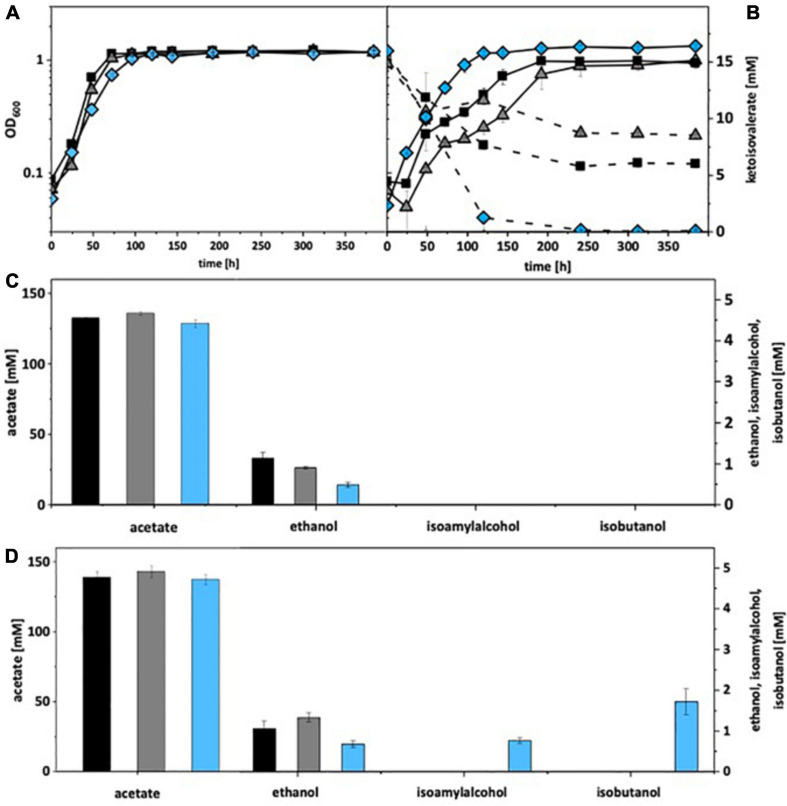
Autotrophic isobutanol production with recombinant *A. woodii* strains by the KivD pathway. Growth behavior **(A,B)**; ketoisovalerate consumption **(B)**; product pattern **(C,D)**. *A. woodii* [WT], black, squares; *A. woodii* [pM83], gray, triangles; *A. woodii* [pKAIA], blue, diamonds. Panels **(A,C)** without ketoisovalerate supplementation; Panels **(B,D)** with ketoisovalerate supplementation. Each strain was analyzed in biological triplicates (*n* = 3).

**FIGURE 5:**
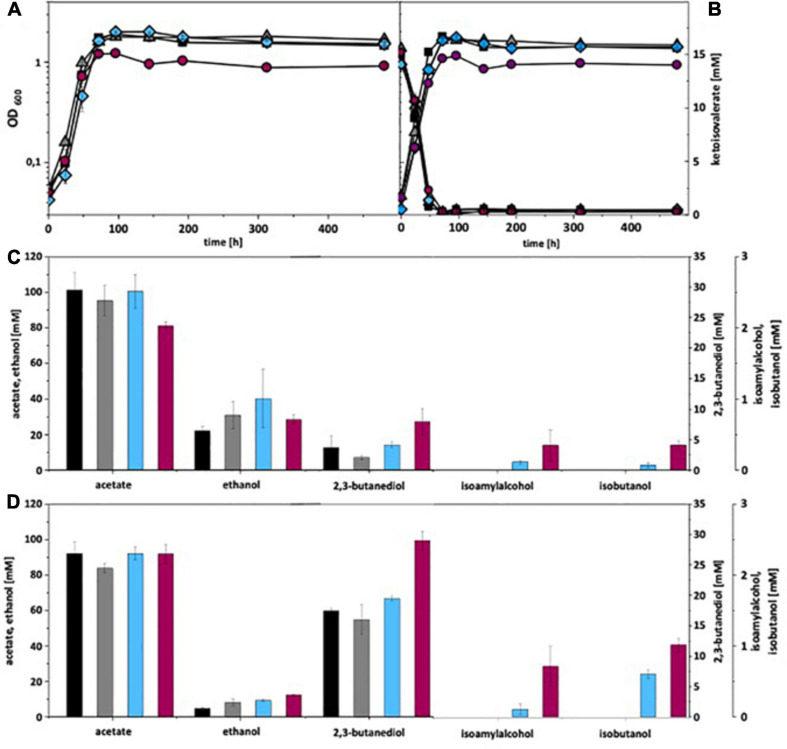
Autotrophic isobutanol production with recombinant *C. ljungdahlii* strains by the KivD pathway. Growth behavior **(A,B)**; ketoisovalerate consumption **(B)**; product pattern **(C,D)**. *C. ljungdahlii* [WT], black, squares; *C. ljungdahlii* [pM83], gray, triangles; *C. ljungdahlii* [pKAIA], blue, diamonds; *C. ljungdahlii:ilvE* [pKAIA], purple, circles; Panels **(A,C)** without ketoisovalerate supplementation; Panels **(B,D)** with ketoisovalerate supplementation. Each strain was analyzed in biological triplicates (*n* = 3).

Under autotrophic growth conditions, recombinant *A. woodii* strains formed no isobutanol. With addition of 15 mM isovalerate, *A. woodii*[pKAIA] produced 1.7 mM isobutanol and 0.8 mM isoamyl alcohol (Figure 4). The respective data for *C. ljungdahlii* are shown in Figure 5. Only *C. ljungdahlii*[pKAIA] and *C. ljungdahlii:ilvE*[pKAIA] formed isobutanol (and isoamyl alcohol) without ketoisovalerate addition. The latter strain formed three times more acohols (up to 0.4 mM isobutanol). Addition of ketoisovalerate increased production of both alcohols in the pKAIA-carrying strains (up to 1 mM in *C. ljungdahlii:ilvE*[pKAIA]).

### Coenzyme Dependency of Isobutanol Production *via* Ketoisovalerate Decarboxylase (KivD)

The *ilvC* gene product of *C. ljungdahlii*, the ketol-acid reductoisomerase, uses NADPH for reduction of acetolactate to 2,3-dihydroxyisovalerate. However, NADPH might be a limiting factor in a catabolic pathway with high amounts of products. Therefore, this gene in pKAIA was replaced by a similar gene encoding a NADH-dependent IlvC enzyme from *E. coli* (Brinkmann-Chen et al., 2013). The nucleotide sequence of Ec_IlvC^*P2D1–A1*^ was codon-optimized for clostridia, PCR-amplified, and subcloned into pKAIA by replacing the *C. ljungdahlii ilvC* gene. The resulting plasmid was designated pKAI_NADH_A (Supplementary Figure 6). This plasmid was transformed into *C. ljungdahlii:ilvE*, and the recombinant tested in heterotrophic and autotrophic fermentations. With fructose as a carbon source, *C. ljungdahlii:ilvE*[pKAI_NADH_A] produced isobutanol only upon addition of ketoisovalerate. The production was slightly higher than with the corresponding pKAIA-carrying strain (1.6 vs. 1.4 mM). Under autotrophic conditions without isovalerate supplementation, *C. ljungdahlii:ilvE*[pKAI_NADH_A] formed only trace amounts of isobutanol (0.1 mM). Even with isovalerate addition, lower amounts were produced than by *C. ljungdahlii:ilvE*[pKAIA] (0.8 mM compared to 1 mM).

## Discussion

The data presented clearly demonstrate that subcloning of both, the KivD as well as the Kor pathway lead to heterologous expression of isobutanol formation in acetogenic bacteria. Both key enzymes, ketoisovalerate decarboxylase and ketoisovalerate ferredoxin oxidoreductase, belong to the group of pyruvate decarboxylases/pyruvate oxidoreductases and are dependent on thiamine pyrophosphate (Heider et al., 1996; de la Plaza et al., 2004). The active forms are homo- or heterotetramers, respectively. KivD from *L. lactis* decarboxylates preferentially ketoisovalerate, but also showed specific activity toward other keto compounds, from ketoisocaproate (23% relative activity) to pyruvate (0.6% relative activity) (de la Plaza et al., 2004). The temperature optimum is 45°C, but more than 74% relative activity is found between 30 and 50°C (de la Plaza et al., 2004). The pH optimum is more pronounced, with a peak at pH 6.5 and app. 90% relative activity between pH 6 and 7 (de la Plaza et al., 2004). Kor has been purified from *Pyrococcus* and *Thermococcus* strains, which are proteolytic and hyperthermophilic archaea. Highest activity was observed with ketoisovalerate, lowest activity with phenylpyruvate, pyruvate, and glyoxylate (Heider et al., 1996). Based on the back reaction of acetyl-CoA + CO_2_ + reduced ferredoxin to pyruvate, Xiong et al. (2016) characterized the *kor* gene clusters of *C. thermocellum* as reversed pyruvate ferredoxin oxidoreductases. *T. litoralis* Kor exhibits a sharp pH optimum at 7, with less than 50% relative activity of pH 6 and 25% at pH 8 (Heider et al., 1996). The temperature optimum of enzymes from the different archaea varies between 90 and 98°C (Heider et al., 1996).

Our results demonstrate that the *kor3* and *kor2* gene clusters are the best suited candidates for further improvement of isobutanol production in acetogens by the Kor pathway, especially in *A. woodii*. Interestingly, the relatively low similarity between the respective gene products (Supplementary Figure 7) does not show a massive effect on product formation. Although the titers are still low (0.1 mM without and up to 2.9 mM with ketoisovalerate addition), it must be kept in mind that in other reported production organisms such as *E. coli* and *C. glutamicum* only numerous additional gene inactivations finally led to promising strains (Atsumi et al., 2008; Blombach et al., 2011). The formation of isoamyl alcohol (3-methyl-1-butanol) is most probably due to the relatively broad substrate spectrum of ketoisovalerate ferredoxin oxidoreductase (and also ketoisovalerate decarboxylase) (Heider et al., 1996; Atsumi et al., 2008). Ketoisocaproate would be the precursor and is metabolized by both enzymes. This substance is formed naturally during leucine biosynthesis. Interestingly, the introduction of the *kor1* and *kor2* gene clusters led in both, *A. woodii* and *C. ljungdahlii* to an app. doubling of the ethanol formation, but only under autotrophic conditions upon addition of ketoisovalerate. *kor2* in *A. woodii* under heterotrophic conditions resulted in the same phenomenon, whereas in *C. ljungdahlii* rather a decrease in ethanol formation in *kor*-carrying strains under heterotrophic conditions could be observed. The reason for this phenomenon is not obvious, as especially in *A. woodii* the stoichiometry of added ketoisovalerate and formed ethanol does not match. On the other hand, the increase of 2,3-butanediol production in all *C. ljungdahlii* strains upon addition of ketoisovalerate can be easily explained. Obviously, ketoisovalerate was converted to acetolactate, which was then decarboxylated and reduced to yield 2,3-butanediol.

The KivD pathway also allowed formation of trace amounts of isobutanol in *A. woodii* (heterotrophic conditions) and *C. ljungdahlii* (autotrophic conditions). Addition of ketoisovalerate clearly stimulated isobutanol production in both organisms under all tested growth conditions. The inactivation of *ilvE* in *C. ljungdahlii* (blocking the last step of valine biosynthesis) led to a significant increase in isobutanol production. This stresses again the necessity of further tailored metabolic mutations for optimizing the isobutanol fermentation. Unfortunately, an attempt during this project to inactivate the *aldC* gene of *C. ljungdahlii* (encoding the acetolactate decarboxylase, which catalyzes the first step of 2,3-butanediol synthesis) failed. Such a mutant would have prevented the carbon flow toward 2,3-butanediol. It was also disappointing that changing the coenzyme specificity of IlvC, the ketol-acid reductoisomerase, of *C. ljungdahlii* from NADPH to NADH did not result in an increase in isobutanol formation. However, as already stressed before, most probably only a combination of a series of metabolic alterations will result in a suitable production strain, as was the case with other heterotrophic bacteria.

What targets for such mutations can be imagined? Of course, reduction of other products will be the primary goal. Acetate formation is of vital importance for acetogens regarding ATP formation, but it can be further converted to ethanol using aldehyde ferredoxin oxidoreductase and alcohol dehydrogenase. The required reducing equivalents (reduced ferredoxin and NADH) can be easily generated from hydrogen under autotrophic conditions. Thus, 2,3-butanediol is the prime candidate for a product to be eliminated. If the ClosTron technology does not work (as mentioned, our attempt to inactivate the *ilvC* gene failed), CRISPR/Cas9-based genome editing will be an alternative that has recently been developed for *C. ljungdahlii* (Huang et al., 2016; Jin et al., 2020). The optimal temperatures for both key enzymes, ketoisovalerate decarboxylase (45°C) and ketoisovalerate ferredoxin oxidoreductase (growth temperature of *C. thermocellum* is 60°C), significantly exceed the range of mesophilic fermentations. The same applies to the pH optimum of both enzymes. Acetogenic bacteria generate a low pH, which results in reduced enzyme activity. Also, the broad enzyme substrate range is a disadvantage, as is obvious from the formation of isoamyl alcohol by *A. woodii*. Thus, mutational adaptation of the key enzymes with respect to pH and temperature tolerance as well as increase of substrate specificity will be another prime target in future. The change of coenzyme specificity from NADPH to NADH has already been achieved for IlvC from *C. ljungdahlii* and can thus easily be combined with the aforementioned alterations. The benefit of a NADH-depending alcohol dehydrogenase replacement in the natural, decarboxylase-based isobutanol formation pathway of Shimwellia blattae has recently been shown by Acedos et al. (2021). Finally, growing the strains in gassed and stirred bioreactors will certainly improve the fermentation outcome. In fact, a *C. ljungdahlii:ilvE* strain carrying pKAIA showed under such conditions an increased isobutanol production by factor 6.5 (Hermann et al., 2021).

Further metabolic engineering will focus on the *kor3* gene cluster in case of *A. woodii* and the KivD pathway in *C. ljungdahlii:ilvE*[pKAIA], which so far allowed highest product formation. Strains suitably improved as described above might then become a viable option for commercial isobutanol synthesis from waste and greenhouse gases such as CO and CO_2_.

## Data Availability Statement

The original contributions presented in the study are included in the article/Supplementary Material, further inquiries can be directed to the corresponding author/s.

## Author Contributions

SW, FB, MH, RT, and PD conceived and designed the experiments. SW and SL performed the experiments. SW, MH, SL, FB, RT, and PD analyzed the data. MH, SL, FB, RT, and PD wrote the manuscript. All authors contributed to the article and approved the submitted version.

## Conflict of Interest

The authors declare that the research was conducted in the absence of any commercial or financial relationships that could be construed as a potential conflict of interest.

## Footnotes

1 https://www.dsmz.de/collection/catalog/details/culture/DSM-4553

2 https://www.lgcstandards-atcc.org/products/all/55383.aspx#culturemethod

3 https://international.neb.com/products/e5520-nebuilder-hifi-dna-assembly-cloning-kit#ProductInformation

4 www.clostron.com

## Supplementary Material

Supplementary Figure 1 Schematic representation of plasmid pMTL83151_ptaack_aacht_cac. *adhE2*, bifunctional aldehyde/alcohol dehydrogenase gene (*C. acetobutylicum*); *abfD*, 4-hydroxybutyryl-CoA dehydratase gene (*Clostridium scatologenes*); *crt*, crotonase gene (*C. acetobutylicum*); *hbd*, 3-hydroxybutyryl-CoA dehydrogenase gene (*C. acetobutylicum*); *thlA*, acetyl-CoA acetyltransferase gene (*C. acetobutylicum*); *catP*, chloramphenicol resistance gene; ColE1, origin of replication for Gram-negative bacteria; *repH*, origin of replication for Gram-positive bacteria; *lacZ alpha*, truncated β-galactosidase gene (*E. coli*); *traJ*, gene for DNA transfer by conjugation.

Supplementary Figure 2 Schematic representation of plasmids pKOR1, pKOR2, and pKOR3. *kor1*, *kor2, kor3*, potential ketoisovalerate ferredoxin oxidoreductases gene clusters (*C. thermocellum*); *adhE2*, bifunctional aldehyde/alcohol dehydrogenase gene (*C. acetobutylicum*); P*_*pta–ack*_*, promoter upstream of *pta-ack* genes (*C. ljungdahlii*); *catP*, chloramphenicol resistance gene; ColE1, origin of replication for Gram-negative bacteria; *repH*, origin of replication for Gram-positive bacteria; *traJ*, gene for DNA transfer by conjugation.

Supplementary Figure 3 Schematic representation of plasmid pJUL34. P_tuf_, promoter upstream of EF-Tu gene (*L. lactis*); *kivD*, ketoisovalerate decarboxylase gene (*L. lactis*); *adhA*, alcohol dehydrogenase gene (*Corynebacterium glutamicum*); *lacZ alpha*, truncated β-galactosidase gene (*E. coli*); *aph3*, kanamycin resistance gene; *sacB*, levansucrase gene; repBL1, origin of replication for *Corynebacterium*.

Supplementary Figure 4 Schematic representation of plasmid pKAIA. *kivD*, ketoisovalerate decarboxylase gene (*L. lactis*); *adhA*, alcohol dehydrogenase gene (*C. glutamicum*); *ilvC*, ketol-acid reductoisomerase gene (*C. ljungdahlii*); *ilvD*, dihydroxy-acid dehydratase gene (*C. ljungdahlii*); *alsS*, acetolactate synthase gene (*C. ljungdahlii*); P*_*pta–ack*_*, promoter upstream of *pta-ack* genes (*C. ljungdahlii*); *catP*, chloramphenicol resistance gene; ColE1, origin of replication for Gram-negative bacteria; *repH*, origin of replication for Gram-positive bacteria; *traJ*, gene for DNA transfer by conjugation.

Supplementary Figure 5 Sequence of the commercially synthesized and codon-optimized *ilvC*^*NADH*^ gene.

Supplementary Figure 6 Schematic representation of plasmid pKAI_NADH_A. *kivD*, ketoisovalerate decarboxylase gene (*L. lactis*); *adhA*, alcohol dehydrogenase gene (*Corynebacterium glutamicum*); *ilvC*, ketol-acid reductoisomerase gene (*C. ljungdahlii*); i*lvC*^*NADH*^, NADH-dependent ketol-acid reductoisomerase gene (Ec_IlvCP2D1-A1 from *E. coli*, codon-optimized for clostridia); *ilvD*, dihydroxy-acid dehydratase gene (*C. ljungdahlii*); *alsS*, acetolactate synthase gene (*C. ljungdahlii*); P*_*pta–ack*_*, promoter upstream of *pta-ack* genes (*C. ljungdahlii*); *catP*, chloramphenicol resistance gene; ColE1, origin of replication for Gram-negative bacteria; *repH*, origin of replication for Gram-positive bacteria; *traJ*, gene for DNA transfer by conjugation.

Supplementary Figure 7 tBLASTx comparison of gene clusters Clo1313_0020-0023 [*kor1*], Clo1313_0382-0385 [*kor2*], and Clo1313_1353-1356 [*kor3*] from *C. thermocellum*.

Supplementary Figure 8 Heterotrophic isobutanol production with recombinant *C. ljungdahlii* strains by the Kor pathway. Growth behavior **(A,D)**; fructose consumption **(B,E)**; ketoisovalerate consumption **(E)**; product pattern **(C,F)**. *C. ljungdahlii* [WT], black, squares; *C. ljungdahlii* [pM83], gray, triangles; *C. ljungdahlii* [pKOR1], green, diamonds; *C. ljungdahlii* [pKOR2], light green, circles; *C. ljungdahlii* [pKOR3], dark green, triangle pointing downward. Panels **(A–C)** without ketoisovalerate supplementation; Panels **(D–F)** with ketoisovalerate supplementation. Each strain was analyzed in biological triplicates (*n* = 3).

Supplementary Figure 9 Autotrophic isobutanol production with recombinant *C. ljungdahlii* strains by the Kor pathway. Growth behavior **(A,B)**; ketoisovalerate consumption **(B)**; product pattern **(C,D)**. *C. ljungdahlii* [WT], black, squares; *C. ljungdahlii* [pM83], gray, triangles; *C. ljungdahlii* [pKOR1], green, diamonds; *C. ljungdahlii* [pKOR2], light green, circles; *C. ljungdahlii* [pKOR3], dark green, triangle pointing downward. Panels **(A,C)** without ketoisovalerate supplementation; Panels **(B,D)** with ketoisovalerate supplementation. Each strain was analyzed in biological triplicates (*n* = 3).

Supplementary Figure 10 Heterotrophic isobutanol production with recombinant *A. woodii* strains by the KivD pathway. Growth behavior **(A,D)**; fructose consumption **(B,E)**; ketoisovalerate consumption **(E)**; product pattern **(C,F)**. *A. woodii* [WT], black, squares; *A. woodii* [pM83], gray, triangles; *A. woodii* [pKAIA], blue, diamonds. Panels **(A–C)** without ketoisovalerate supplementation; Panels **(D–F)**, with ketoisovalerate supplementation. Each strain was analyzed in biological triplicates (*n* = 3).

Supplementary Figure 11 Heterotrophic isobutanol production with recombinant *C. ljungdahlii* strains by the KivD pathway. Growth behavior **(A,D)**; fructose consumption **(B,E)**; ketoisovalerate consumption **(E)**; product range **(C,F)**. *C. ljungdahlii* [WT], black, squares; *C. ljungdahlii* [pKAIA], blue, diamonds; *C. ljungdahlii:ilvE* [pKAIA], purple, circles. Panels **(A–C)** without ketoisovalerate supplementation; Panels **(D–F)** with ketoisovalerate supplementation. Each strain was analyzed in biological triplicates (*n* = 3).

## References

[B1] Acedos M. G. de la Torre I. Santos V. E. García-Ochoa F. García J. L. Galán B. (2021). Modulating redox metabolism to improve isobutanol production in *Shimwellia blattae*. *Biotechnol. Biofuels* 14 1–11. 10.1186/s13068-020-01862-1 33407735 PMC7789792

[B2] Atsumi S. Hanai T. Liao J. C. (2008). Non-fermentative pathways for synthesis of branched-chain higher alcohols as biofuels. *Nature* 451 86–89. 10.1038/nature06450 18172501

[B3] Balch W. E. Schoberth S. Tanner R. S. Wolfe R. S. (1977). *Acetobacterium*, a new genus of hydrogen-oxidizing, carbon dioxide-reducing, anaerobic bacteria. *Int. J. Syst. Bacteriol.* 27 355–361.

[B4] Black W. B. Zhang L. Kamoku C. Liao J. C. Li H. (2018). Rearrangement of coenzyme A-acylated carbon chain enables synthesis of isobutanol via a novel pathway in *Ralstonia eutropha*. *ACS Synth. Biol.* 7 794–800. 10.1021/acssynbio.7b00409 29429336

[B5] Blombach B. Riester T. Wieschalka S. Ziert C. Youn J.-W. Wendisch V. F. (2011). *Corynebacterium glutamicum* tailored for efficient isobutanol production. *Appl. Environ. Microbiol.* 77 3300–3310. 10.1128/AEM.02972-10 21441331 PMC3126470

[B6] Brigham C. (2019). Perspectives for the biotechnological production of biofuels from CO_2_ and H_2_ using *Ralstonia eutropha* and other ‘Knallgas’ bacteria. *Appl. Microbiol. Biotechnol.* 103 2113–2120. 10.1007/s00253-019-09636-y 30666363

[B7] Brinkmann-Chen S. Flock T. Cahn J. K. B. Snow C. D. Brustad E. M. McIntosh J. A. (2013). General approach to reversing ketol-acid reductoisomerase cofactor dependence from NADPH to NADH. *Proc. Natl. Acad. Sci. U.S.A.* 110 10946–10951. 10.1073/pnas.1306073110 23776225 PMC3704004

[B8] Chen C.-T. Liao J. C. (2016). Frontiers in microbial 1-butanol and isobutanol production. *FEMS Microbiol. Lett.* 363:fnw020. 10.1093/femsle/fnw020 26832641

[B9] de la Plaza M. de Palencia P. F. Peláez C. Requena T. (2004). Biochemical and molecular characterization of α-ketoisovalerate decarboxylase, an enzyme involved in the formation of aldehydes from amino acids by *Lactococcus lactis*. *FEMS Microbiol. Lett.* 238 367–374. 10.1016/j.femsle.2004.07.057 15358422

[B10] Green M. Sambrook J. (2012). *Molecular Cloning: a Laboratory Manual.* Cold Spring Harbor, NY: Cold Spring Harbor Laboratory Press.

[B11] Heap J. T. Kuehne S. A. Ehsaan M. Cartman S. T. Cooksley C. M. Scott J. C. (2010). The ClosTron: mutagenesis in *Clostridium* refined and streamlined. *J. Microbiol. Methods* 80 49–55. 10.1016/j.mimet.2009.10.018 19891996

[B12] Heap J. T. Pennington O. J. Cartman S. T. Carter G. P. Minton N. P. (2007). The ClosTron: a universal gene knock-out system for the genus *Clostridium*. *J. Microbiol. Methods* 70 452–464. 10.1016/j.mimet.2007.05.021 17658189

[B13] Heap J. T. Pennington O. J. Cartman S. T. Minton N. P. (2009). A modular system for *Clostridium* shuttle plasmids. *J. Microbiol. Methods* 78 79–85. 10.1016/j.mimet.2009.05.004 19445976

[B14] Heider J. Mai X. Adams M. W. W. (1996). Characterization of 2-ketoisovalerate ferredoxin oxidoreductase, a new and reversible coenzyme A-dependent enzyme involved in peptide fermentation by hyperthermophilic archaea. *J. Bacteriol.* 178 780–787. 8550513 10.1128/jb.178.3.780-787.1996 PMC177725

[B15] Hermann M. Teleki A. Weitz S. Niess A. Freund A. Bengelsdorf F. R. (2021). Debottlenecking autotrophic isobutanol formation in recombinant *C. ljungdahlii* by systemic analysis. *Front. Bioeng. Biotechnol.* 9:647853. 10.3389/fbioe.2021.647853 33748092 PMC7968104

[B16] Huang H. Chai C. Li N. Rowe P. Minton N. P. Yang S. (2016). CRISPR/Cas9-based efficient genome editing in *Clostridium ljungdahlii*, an autotrophic gas-fermenting bacterium. *ACS Synth. Biol.* 5 1355–1361. 10.1021/acssynbio.6b00044 27276212

[B17] Inoue H. Nojima H. Okayama H. (1990). High efficiency transformation of *Escherichia coli* with plasmids. *Gene* 96 23–28. 10.1016/0378-1119(90)90336-p 2265755

[B18] Jin S. Bae J. Song Y. Pearcy N. Shin J. Kang S. (2020). Synthetic biology on acetogenic bacteria for highly efficient conversion of C1 gases to biochemicals. *Int. J. Mol. Sci.* 21:7639. 10.3390/ijms21207639 33076477 PMC7589590

[B19] Leang C. Ueki T. Nevin K. P. Lovley D. R. (2013). A genetic system for *Clostridium ljungdahlii*: a chassis for autotrophic production of biocommodities and a model homoacetogen. *Appl. Environ. Microbiol.* 79 1102–1109. 10.1128/AEM.02891-12 23204413 PMC3568603

[B20] Li H. Opgenorth P. H. Wernick D. G. Rogers S. Wu T.-Y. Higashide W. (2012). Integrated electromicrobial conversion of CO_2_ to higher alcohols. *Science* 335:1596. 10.1126/science.1217643 22461604

[B21] Lin P. P. Mi L. Morioka A. H. Yoshino K. M. Konishi S. Xu S. C. (2015). Consolidated bioprocessing of cellulose to isobutanol using *Clostridium thermocellum*. *Metab. Eng.* 31 44–52. 10.1016/j.ymben.2015.07.001 26170002

[B22] Lin P. P. Rabe K. S. Takasumi J. L. Kadisch M. Arnold F. H. Liao J. C. (2014). Isobutanol production at elevated temperatures in thermophilic *Geobacillus thermoglucosidasius*. *Metab. Eng.* 24 1–8. 10.1016/j.ymben.2014.03.006 24721011

[B23] Miao R. Xie H. Lindblad P. (2018). Enhancement of photosynthetic isobutanol production in engineered cells of *Synechocystis* PCC 6803. *Biotechnol. Biofuels* 11:267. 10.1186/s13068-018-1268-8 30275907 PMC6158846

[B24] Perutka J. Wang W. Goerlitz D. Lambowitz A. M. (2004). Use of computer-designed group II introns to disrupt *Escherichia coli* DExH/D-box protein and DNA helicase genes. *J. Mol. Biol.* 336 421–439. 10.1016/j.jmb.2003.12.009 14757055

[B25] Tanner R. S. Miller L. M. Yang D. (1993). *Clostridium ljungdahlii* sp. nov., an acetogenic species in clostridial rRNA homology group I. *Int. J. Syst. Bacteriol.* 43, 232–236. 10.1099/00207713-43-2-232 7684239

[B26] Wess J. Brinek M. Boles E. (2019). Improving isobutanol production with the yeast *Saccharomyces cerevisiae* by successively blocking competing metabolic pathways as well as ethanol and glycerol formation. *Biotechnol. Biofuels* 12:173. 10.1186/s13086-019-1486-8 PMC6604370 31303893

[B27] Xiong W. Lin P. P. Magnusson L. Warner L. Liao J. C. Maness P.-C. (2016). CO_2_-fixing one-carbon metabolism in a cellulose-degrading bacterium *Clostridium thermocellum*. *Proc. Natl. Acad. Sci. U.S.A.* 113 13180–13185. 10.1073/pnas.1605482113 27794122 PMC5135332

