# Supplementary figures and images for: Isobutanol Production by Autotrophic Acetogenic Bacteria
Source: Front Bioeng Biotechnol. 2021 Apr 12;9:657253. doi: 10.3389/fbioe.2021.657253 (PMC8072342; doi:10.3389/fbioe.2021.657253)

## Slide 1
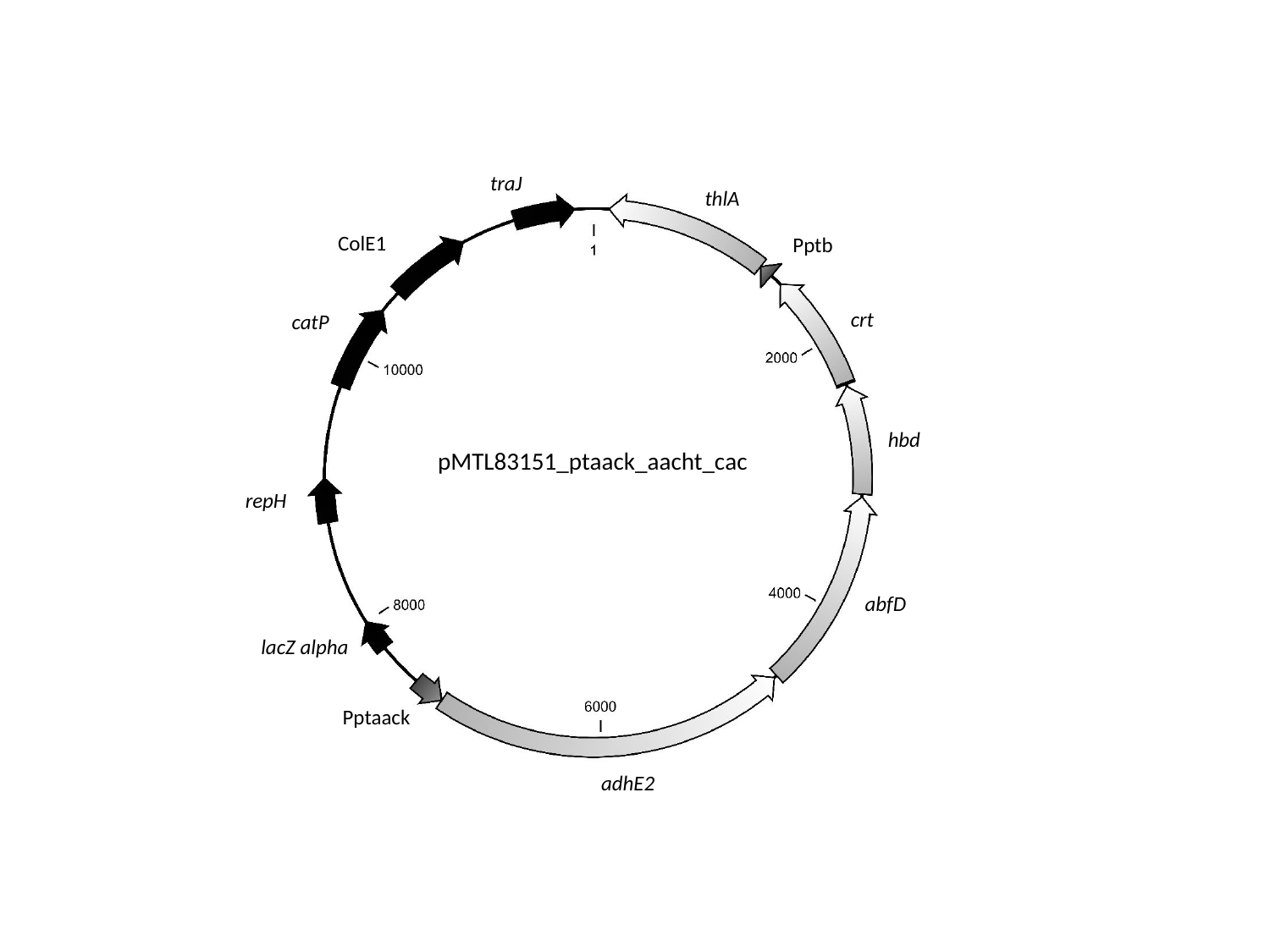

traJ
thlA
ColE1
Pptb
crt
catP
hbd
pMTL83151_ptaack_aacht_cac
repH
abfD
lacZ alpha
Pptaack
adhE2

Supplement: Supplementary Figure 1 — Schematic representation of plasmid pMTL83151_ptaack_aacht_cac. adhE2, bifunctional aldehyde/alcohol dehydrogenase gene (C. acetobutylicum); abfD, 4-hydroxybutyryl-CoA dehydratase gene (Clostridium scatologenes); crt, crotonase gene (C. acetobutylicum); hbd, 3-hydroxybutyryl-CoA dehydrogenase gene (C. acetobutylicum); thlA, acetyl-CoA acetyltransferase gene (C. acetobutylicum); catP, chloramphenicol resistance gene; ColE1, origin of replication for Gram-negative bacteria; repH, origin of replication for Gram-positive bacteria; lacZ alpha, truncated β-galactosidase gene (E. coli); traJ, gene for DNA transfer by conjugation. [file Presentation_1.PPTX]

## Slide 1
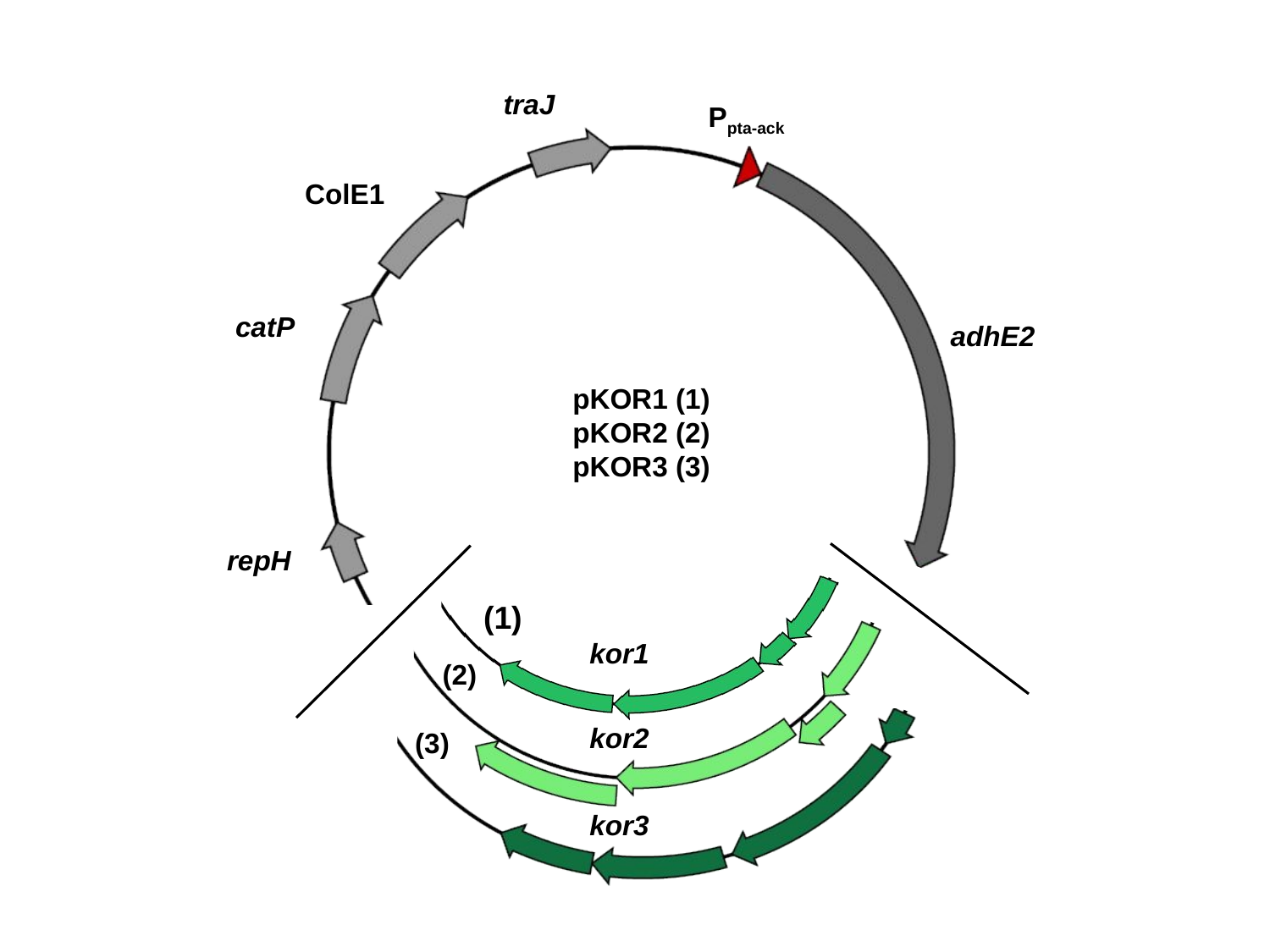

traJ
Ppta-ack
ColE1
catP
adhE2
pKOR1 (1)
pKOR2 (2)
pKOR3 (3)
repH
(1)
kor1
(2)
kor2
(3)
kor3

Supplement: Supplementary Figure 2 — Schematic representation of plasmids pKOR1, pKOR2, and pKOR3. kor1, kor2, kor3, potential ketoisovalerate ferredoxin oxidoreductases gene clusters (C. thermocellum); adhE2, bifunctional aldehyde/alcohol dehydrogenase gene (C. acetobutylicum); Ppta–ack, promoter upstream of pta-ack genes (C. ljungdahlii); catP, chloramphenicol resistance gene; ColE1, origin of replication for Gram-negative bacteria; repH, origin of replication for Gram-positive bacteria; traJ, gene for DNA transfer by conjugation. [file Presentation_2.PPTX]

## Slide 1
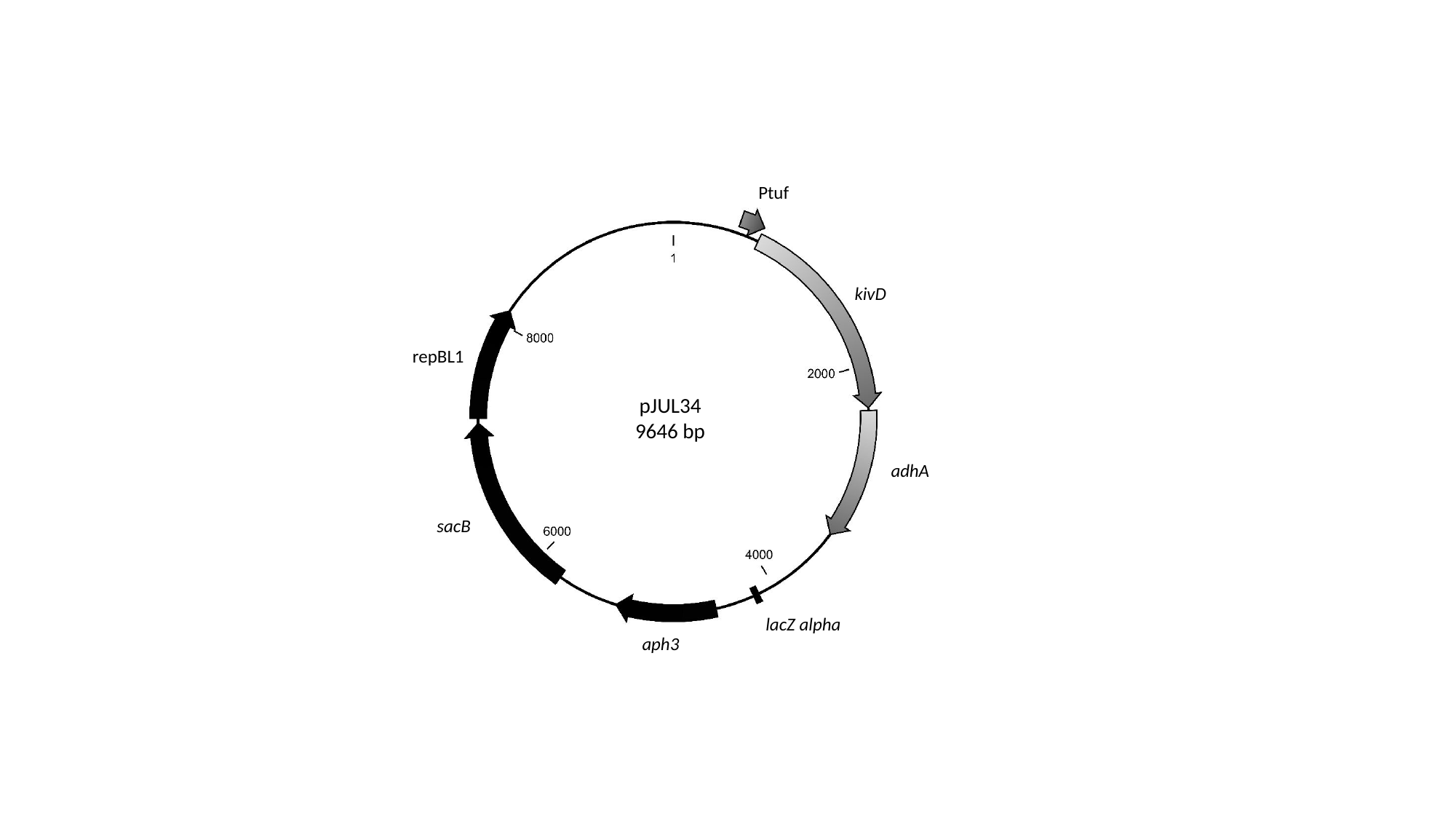

Ptuf
kivD
repBL1
pJUL34
9646 bp
adhA
sacB
lacZ alpha
aph3

Supplement: Supplementary Figure 3 — Schematic representation of plasmid pJUL34. Ptuf, promoter upstream of EF-Tu gene (L. lactis); kivD, ketoisovalerate decarboxylase gene (L. lactis); adhA, alcohol dehydrogenase gene (Corynebacterium glutamicum); lacZ alpha, truncated β-galactosidase gene (E. coli); aph3, kanamycin resistance gene; sacB, levansucrase gene; repBL1, origin of replication for Corynebacterium. [file Presentation_3.PPTX]

## Slide 1
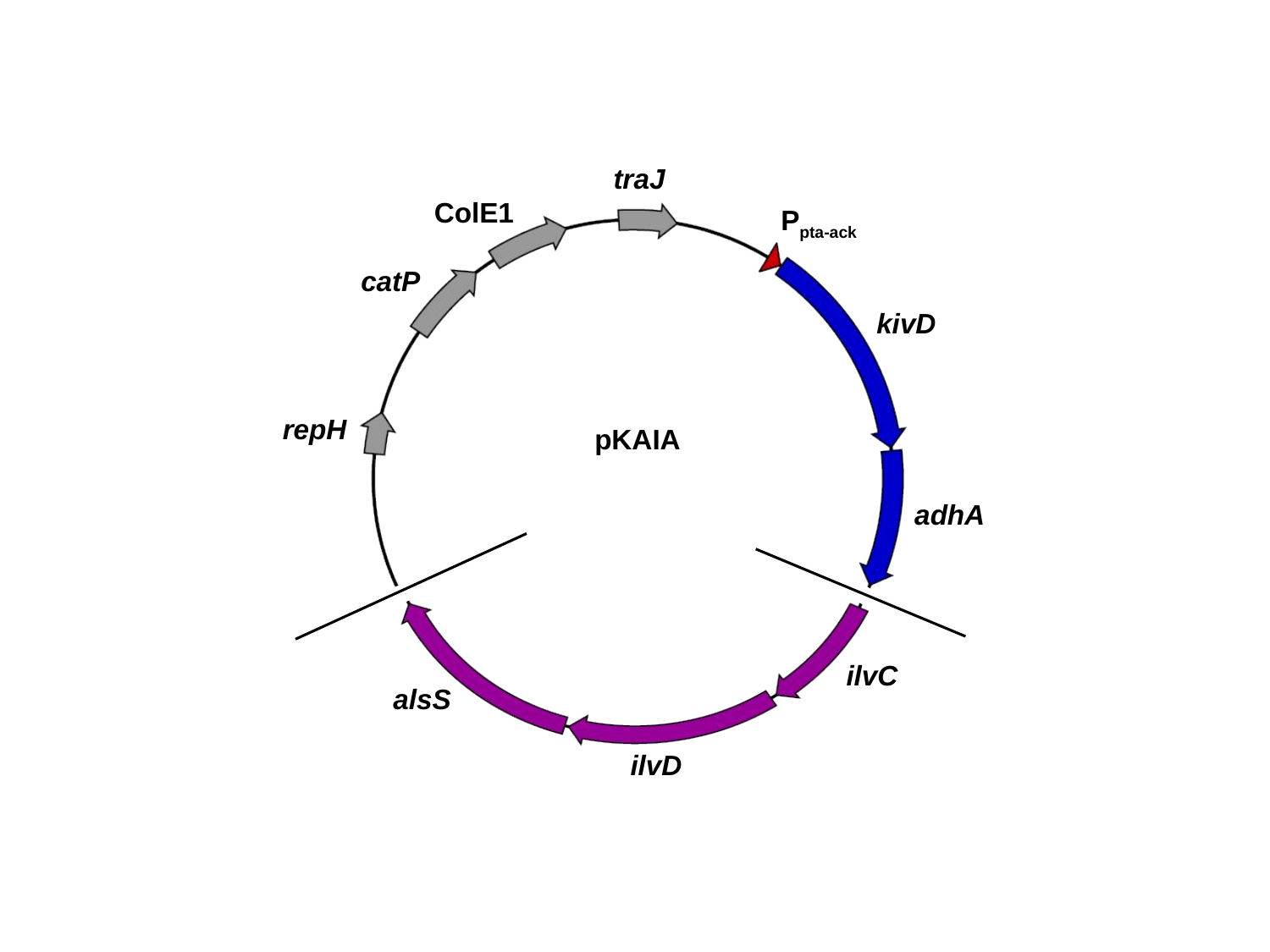

traJ
ColE1
Ppta-ack
catP
kivD
repH
 pKAIA
adhA
ilvC
alsS
ilvD

Supplement: Supplementary Figure 4 — Schematic representation of plasmid pKAIA. kivD, ketoisovalerate decarboxylase gene (L. lactis); adhA, alcohol dehydrogenase gene (C. glutamicum); ilvC, ketol-acid reductoisomerase gene (C. ljungdahlii); ilvD, dihydroxy-acid dehydratase gene (C. ljungdahlii); alsS, acetolactate synthase gene (C. ljungdahlii); Ppta–ack, promoter upstream of pta-ack genes (C. ljungdahlii); catP, chloramphenicol resistance gene; ColE1, origin of replication for Gram-negative bacteria; repH, origin of replication for Gram-positive bacteria; traJ, gene for DNA transfer by conjugation. [file Presentation_4.PPTX]

## Slide 1
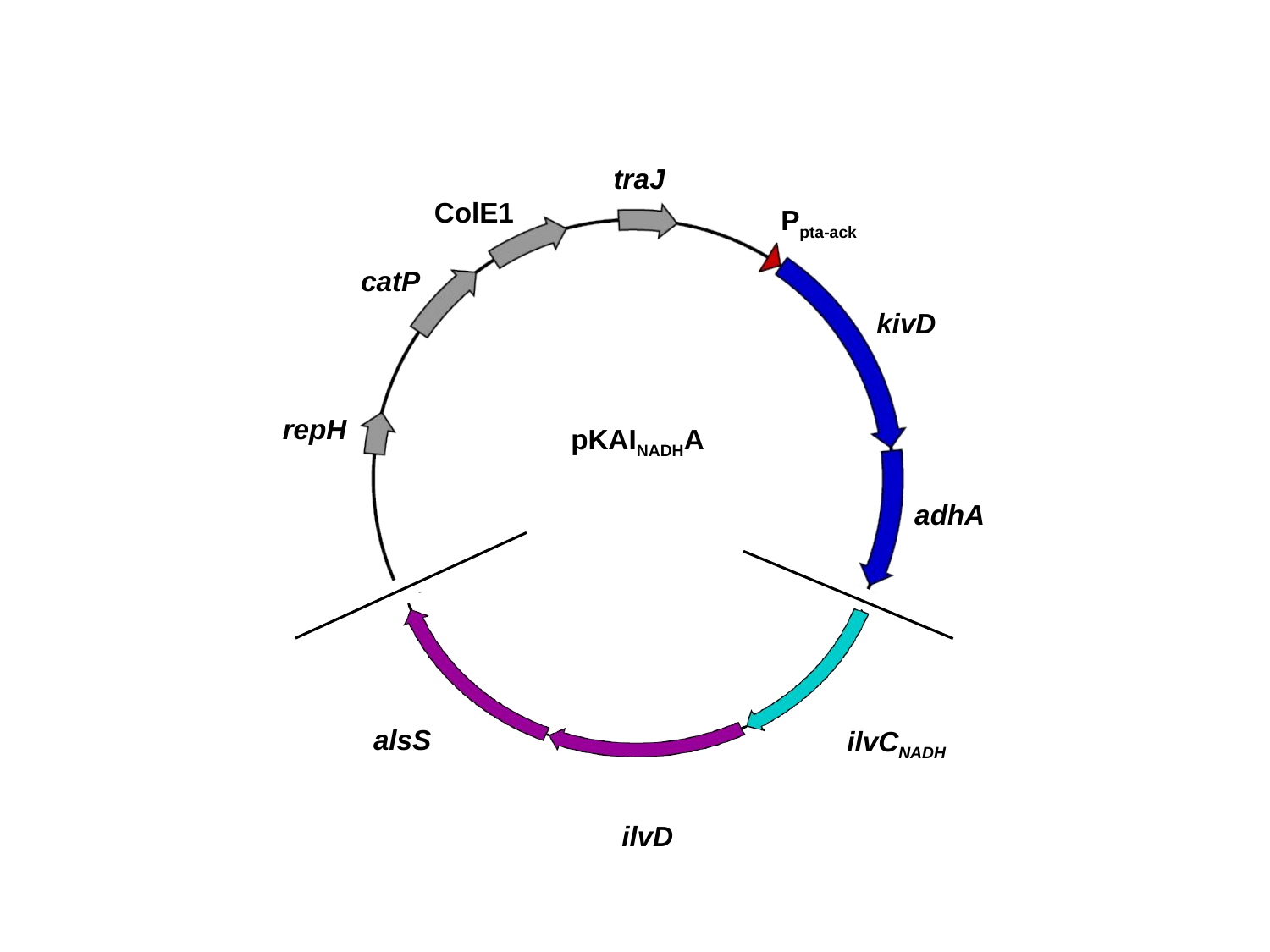

traJ
ColE1
Ppta-ack
catP
kivD
repH
 pKAINADHA
adhA
alsS
ilvCNADH
ilvD

Supplement: Supplementary Figure 6 — Schematic representation of plasmid pKAINADHA. kivD, ketoisovalerate decarboxylase gene (L. lactis); adhA, alcohol dehydrogenase gene (Corynebacterium glutamicum); ilvC, ketol-acid reductoisomerase gene (C. ljungdahlii); ilvCNADH, NADH-dependent ketol-acid reductoisomerase gene (Ec_IlvCP2D1-A1 from E. coli, codon-optimized for clostridia); ilvD, dihydroxy-acid dehydratase gene (C. ljungdahlii); alsS, acetolactate synthase gene (C. ljungdahlii); Ppta–ack, promoter upstream of pta-ack genes (C. ljungdahlii); catP, chloramphenicol resistance gene; ColE1, origin of replication for Gram-negative bacteria; repH, origin of replication for Gram-positive bacteria; traJ, gene for DNA transfer by conjugation. [file Presentation_6.PPTX]

## Slide 1
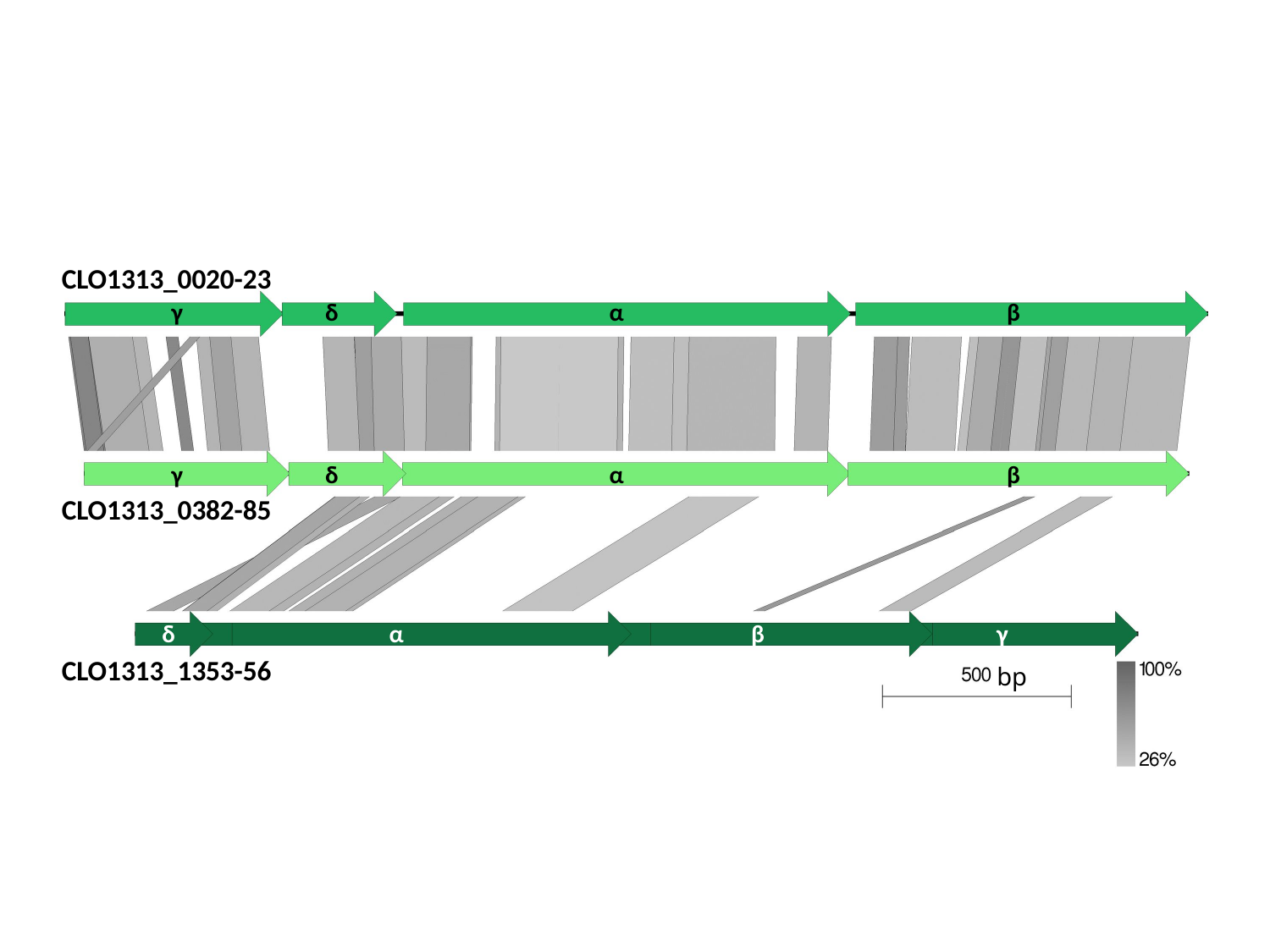

CLO1313_0020-23
γ
δ
α
β
γ
δ
α
β
CLO1313_0382-85
δ
α
β
γ
bp
CLO1313_1353-56

Supplement: Supplementary Figure 7 — tBLASTx comparison of gene clusters Clo1313_0020-0023 [kor1], Clo1313_0382-0385 [kor2], and Clo1313_1353-1356 [kor3] from C. thermocellum. [file Presentation_7.PPTX]

## Slide 1
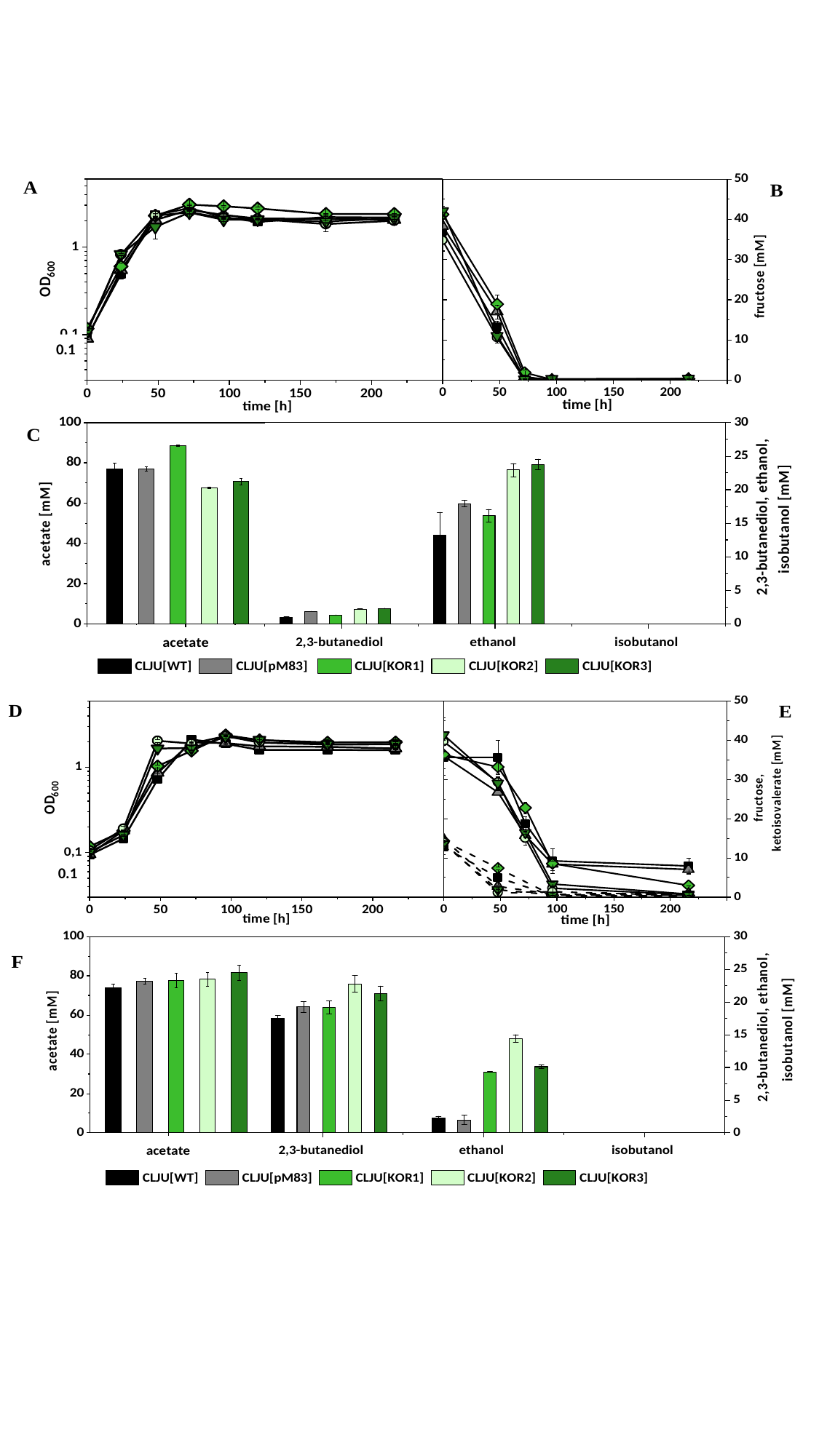

0.1
0.1

Supplement: Supplementary Figure 8 — Heterotrophic isobutanol production with recombinant C. ljungdahlii strains by the Kor pathway. Growth behavior (A,D); fructose consumption (B,E); ketoisovalerate consumption (E); product pattern (C,F). C. ljungdahlii [WT], black, squares; C. ljungdahlii [pM83], gray, triangles; C. ljungdahlii [pKOR1], green, diamonds; C. ljungdahlii [pKOR2], light green, circles; C. ljungdahlii [pKOR3], dark green, triangle pointing downward. Panels (A–C) without ketoisovalerate supplementation; Panels (D–F) with ketoisovalerate supplementation. Each strain was analyzed in biological triplicates (n = 3). [file Presentation_8.PPTX]

## Slide 1
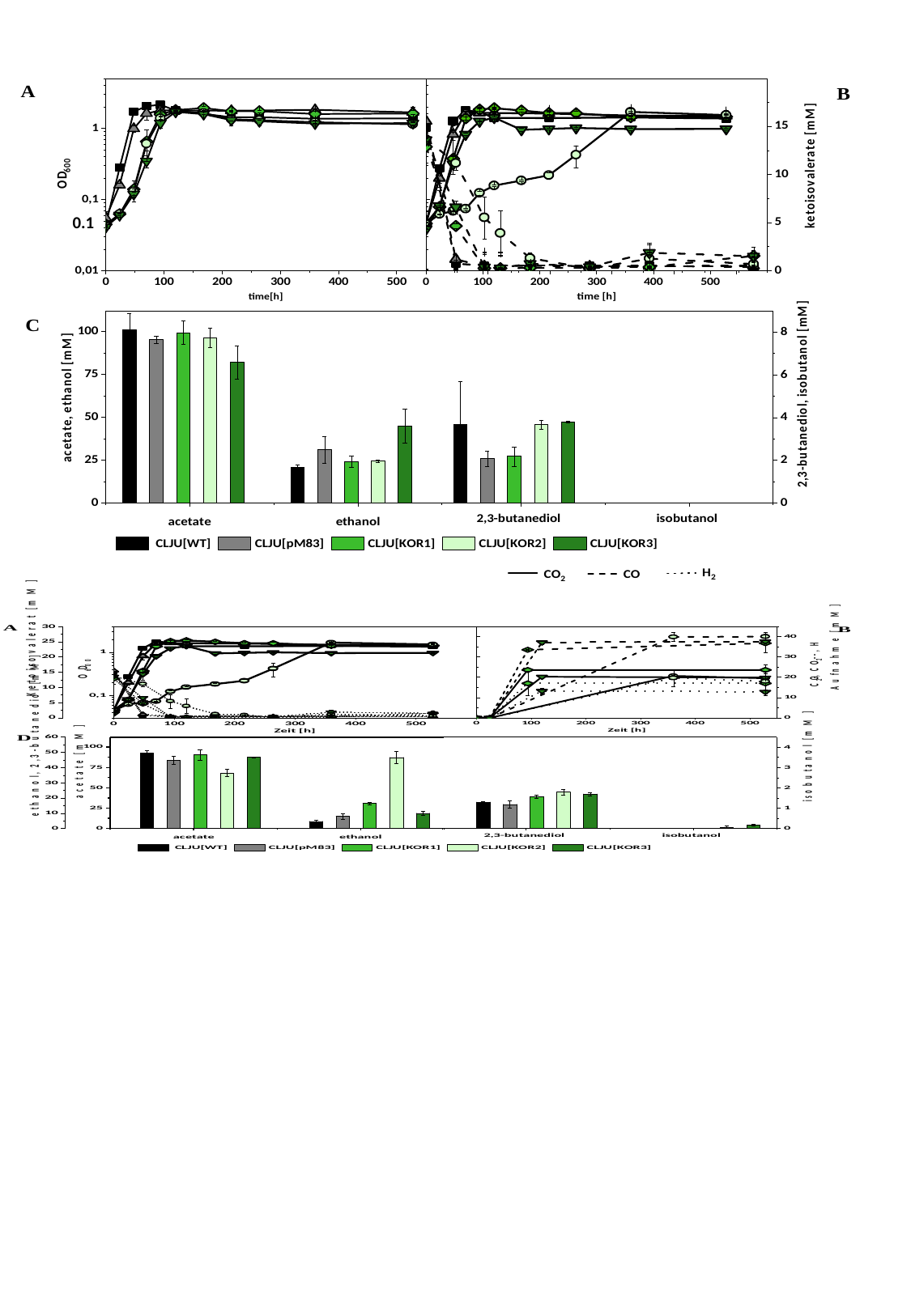

0.1

Supplement: Supplementary Figure 9 — Autotrophic isobutanol production with recombinant C. ljungdahlii strains by the Kor pathway. Growth behavior (A,B); ketoisovalerate consumption (B); product pattern (C,D). C. ljungdahlii [WT], black, squares; C. ljungdahlii [pM83], gray, triangles; C. ljungdahlii [pKOR1], green, diamonds; C. ljungdahlii [pKOR2], light green, circles; C. ljungdahlii [pKOR3], dark green, triangle pointing downward. Panels (A,C) without ketoisovalerate supplementation; Panels (B,D) with ketoisovalerate supplementation. Each strain was analyzed in biological triplicates (n = 3). [file Presentation_9.PPTX]

## Slide 1
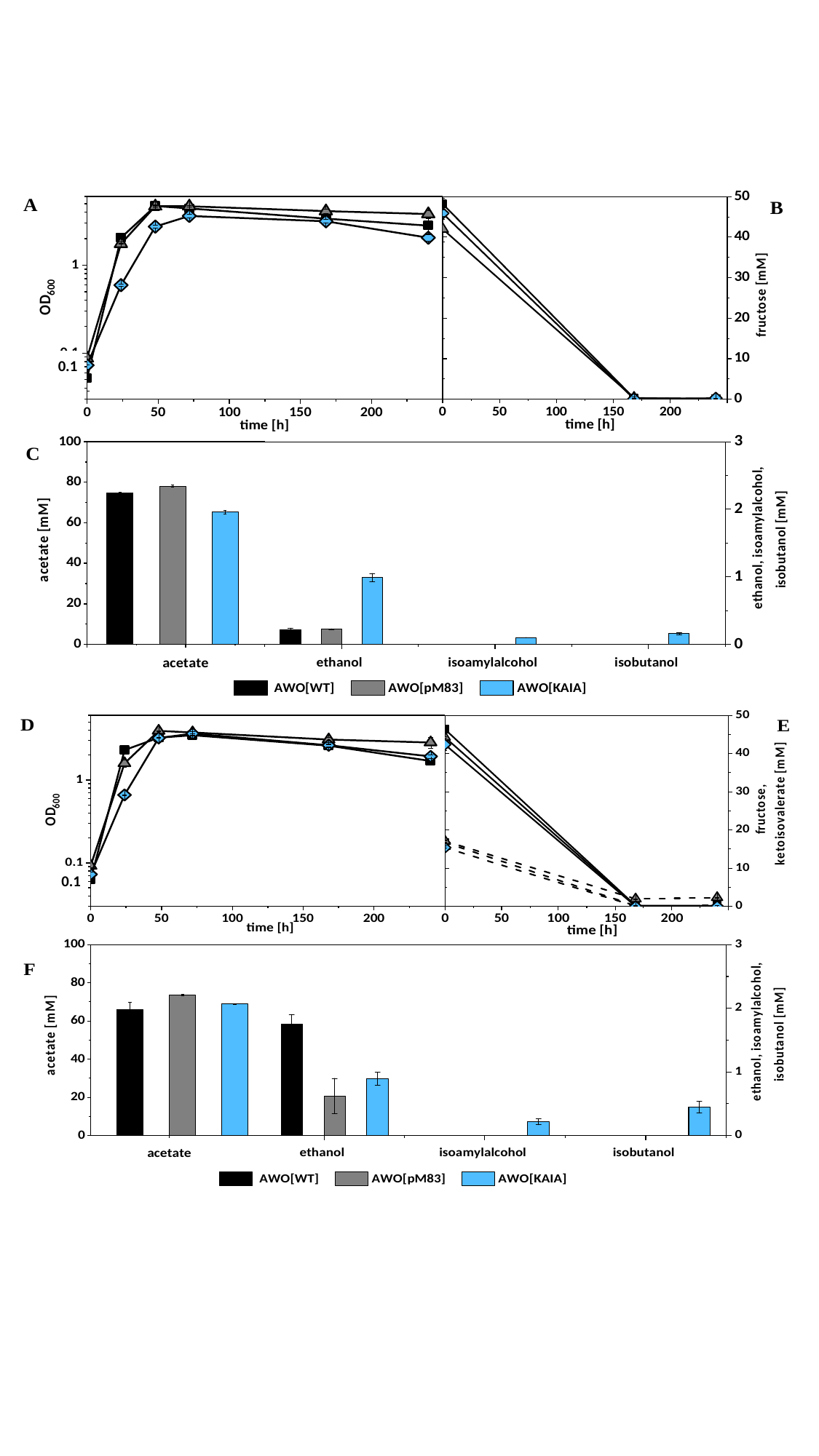

0.1
0.1

Supplement: Supplementary Figure 10 — Heterotrophic isobutanol production with recombinant A. woodii strains by the KivD pathway. Growth behavior (A,D); fructose consumption (B,E); ketoisovalerate consumption (E); product pattern (C,F). A. woodii [WT], black, squares; A. woodii [pM83], gray, triangles; A. woodii [pKAIA], blue, diamonds. Panels (A–C) without ketoisovalerate supplementation; Panels (D–F), with ketoisovalerate supplementation. Each strain was analyzed in biological triplicates (n = 3). [file Presentation_10.PPTX]

## Slide 1
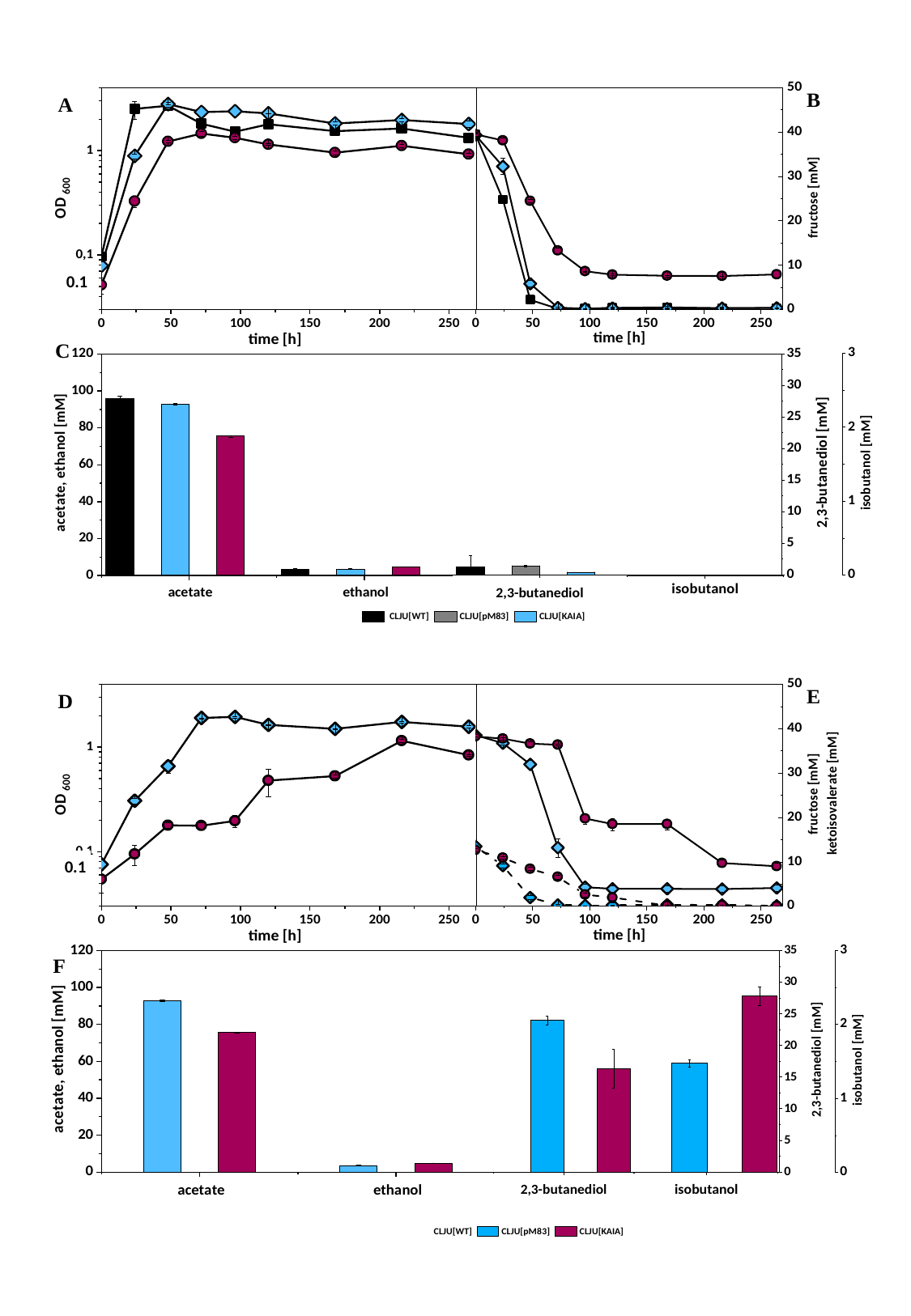

0.1
0.1

Supplement: Supplementary Figure 11 — Heterotrophic isobutanol production with recombinant C. ljungdahlii strains by the KivD pathway. Growth behavior (A,D); fructose consumption (B,E); ketoisovalerate consumption (E); product range (C,F). C. ljungdahlii [WT], black, squares; C. ljungdahlii [pKAIA], blue, diamonds; C. ljungdahlii:ilvE [pKAIA], purple, circles. Panels (A–C) without ketoisovalerate supplementation; Panels (D–F) with ketoisovalerate supplementation. Each strain was analyzed in biological triplicates (n = 3). [file Presentation_11.PPTX]
