# Supplementary material for: Isobutanol Production by Autotrophic Acetogenic Bacteria
Source: Front Bioeng Biotechnol. 2021 Apr 12;9:657253. doi: 10.3389/fbioe.2021.657253 (PMC8072342; doi:10.3389/fbioe.2021.657253)
Supplement: Supplementary Figure 5 — Sequence of the commercially synthesized and codon-optimized ilvCNADH gene. [file Presentation_5.PPTX]

## Slide 1
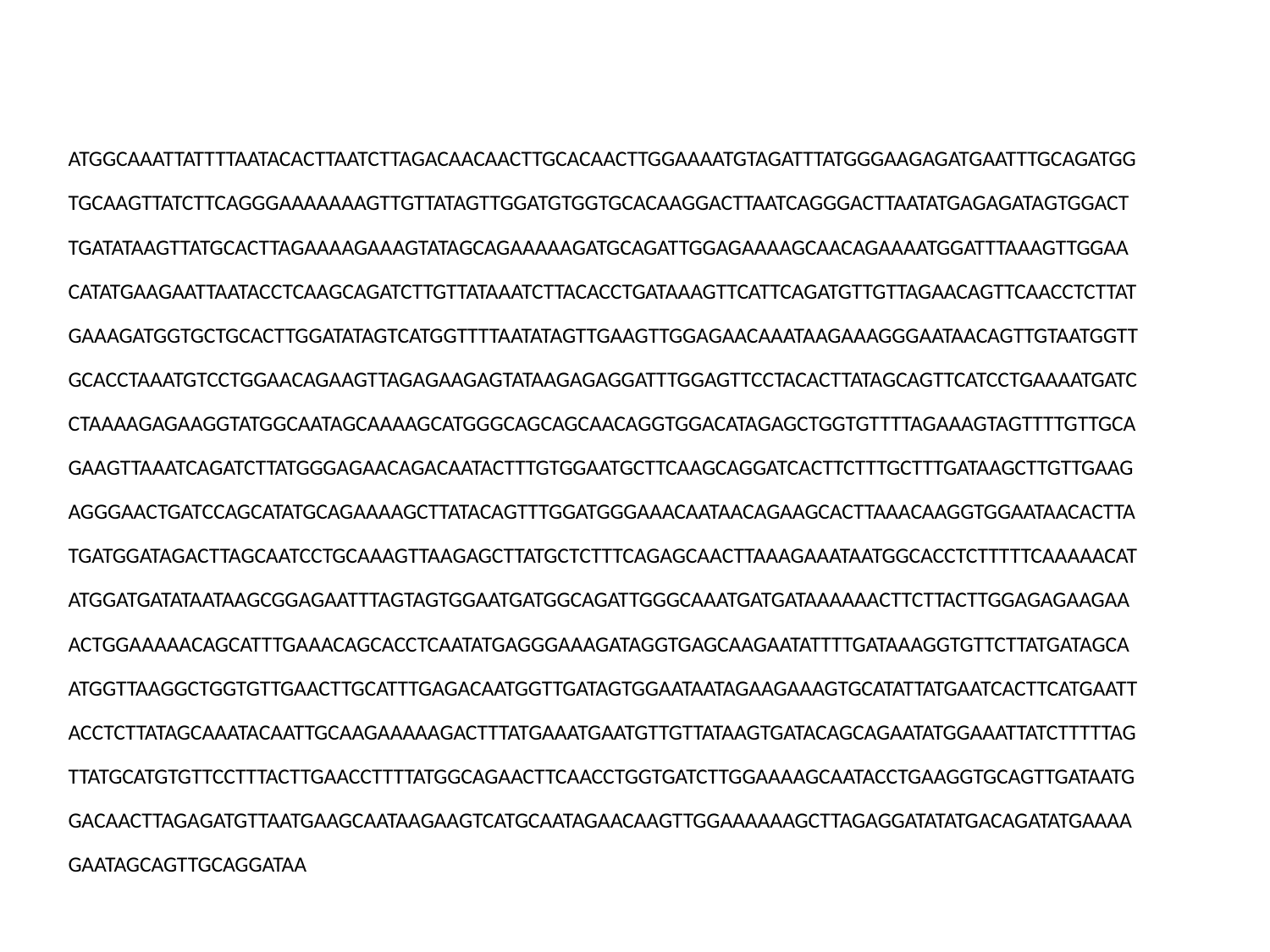

ATGGCAAATTATTTTAATACACTTAATCTTAGACAACAACTTGCACAACTTGGAAAATGTAGATTTATGGGAAGAGATGAATTTGCAGATGGTGCAAGTTATCTTCAGGGAAAAAAAGTTGTTATAGTTGGATGTGGTGCACAAGGACTTAATCAGGGACTTAATATGAGAGATAGTGGACTTGATATAAGTTATGCACTTAGAAAAGAAAGTATAGCAGAAAAAGATGCAGATTGGAGAAAAGCAACAGAAAATGGATTTAAAGTTGGAACATATGAAGAATTAATACCTCAAGCAGATCTTGTTATAAATCTTACACCTGATAAAGTTCATTCAGATGTTGTTAGAACAGTTCAACCTCTTATGAAAGATGGTGCTGCACTTGGATATAGTCATGGTTTTAATATAGTTGAAGTTGGAGAACAAATAAGAAAGGGAATAACAGTTGTAATGGTTGCACCTAAATGTCCTGGAACAGAAGTTAGAGAAGAGTATAAGAGAGGATTTGGAGTTCCTACACTTATAGCAGTTCATCCTGAAAATGATCCTAAAAGAGAAGGTATGGCAATAGCAAAAGCATGGGCAGCAGCAACAGGTGGACATAGAGCTGGTGTTTTAGAAAGTAGTTTTGTTGCAGAAGTTAAATCAGATCTTATGGGAGAACAGACAATACTTTGTGGAATGCTTCAAGCAGGATCACTTCTTTGCTTTGATAAGCTTGTTGAAGAGGGAACTGATCCAGCATATGCAGAAAAGCTTATACAGTTTGGATGGGAAACAATAACAGAAGCACTTAAACAAGGTGGAATAACACTTATGATGGATAGACTTAGCAATCCTGCAAAGTTAAGAGCTTATGCTCTTTCAGAGCAACTTAAAGAAATAATGGCACCTCTTTTTCAAAAACATATGGATGATATAATAAGCGGAGAATTTAGTAGTGGAATGATGGCAGATTGGGCAAATGATGATAAAAAACTTCTTACTTGGAGAGAAGAAACTGGAAAAACAGCATTTGAAACAGCACCTCAATATGAGGGAAAGATAGGTGAGCAAGAATATTTTGATAAAGGTGTTCTTATGATAGCAATGGTTAAGGCTGGTGTTGAACTTGCATTTGAGACAATGGTTGATAGTGGAATAATAGAAGAAAGTGCATATTATGAATCACTTCATGAATTACCTCTTATAGCAAATACAATTGCAAGAAAAAGACTTTATGAAATGAATGTTGTTATAAGTGATACAGCAGAATATGGAAATTATCTTTTTAGTTATGCATGTGTTCCTTTACTTGAACCTTTTATGGCAGAACTTCAACCTGGTGATCTTGGAAAAGCAATACCTGAAGGTGCAGTTGATAATGGACAACTTAGAGATGTTAATGAAGCAATAAGAAGTCATGCAATAGAACAAGTTGGAAAAAAGCTTAGAGGATATATGACAGATATGAAAAGAATAGCAGTTGCAGGATAA
